# Conformational landscape of 2-aminopurine-substituted RNA oligonucleotides from machine-learning-driven enhanced sampling

**DOI:** 10.1039/d6cp01607c

**Published:** 2026-07-10

**Authors:** Revanth Elangovan, Emily Stetson, Julia R. Widom, Dhiman Ray

**Affiliations:** a Department of Chemistry and Biochemistry, University of Oregon, Eugene Oregon-97403 USA jwidom@uoregon.edu dray@uoregon.edu; b Oregon Center for Optical, Molecular and Quantum Science, University of Oregon, Eugene Oregon 97403 USA; c Institute for Molecular Biology, University of Oregon, Eugene Oregon 97403 USA; d Material Science Institute, University of Oregon, Eugene Oregon 97403 USA; e Department of Physics, University of Oregon, Eugene Oregon 97403 USA

## Abstract

Fluorescent base analogs, such as 2-aminopurine (2AP), are frequently used to investigate nucleic acid conformational dynamics, particularly base-stacking interactions, through fluorescence spectroscopy. Although 2AP substitution can induce structural perturbations, properly accounting for these effects enables its use as a probe of complex RNA dynamics. Here, we apply explainable machine-learning-derived surrogate-model collective variables to perform enhanced-sampling simulations that comprehensively explore the free-energy landscapes of both 2AP-substituted and unsubstituted RNA dinucleotides and trinucleotides. Our results show that while 2AP substitution does not significantly alter stacking propensity, it substantially modulates the free energy landscape, leading to a redistribution of populations among stacking modes. The fraction of stacked 2AP conformations closely correlates with experimentally observed “dark state” populations, supporting the hypothesis that base stacking quenches 2AP fluorescence and promotes dark state formation. This work and follow-up studies in larger, physiologically relevant RNA systems will establish the ability to correlate observed emissive or dark states of 2AP with specific structures within the free-energy landscape.

## Abstract 2

Enhanced sampling simulation using Machine-Learning Collective Variables revealed the free-energy landscapes of fluorescent base-analogue-substituted RNA oligonucleotides.

## Graphical abstract

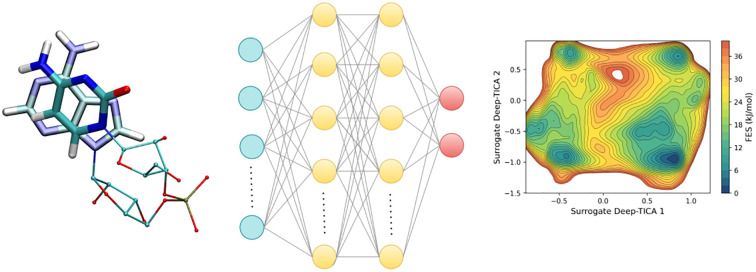

## Introduction

Structural heterogeneity in RNA is foundational for its ability to act as a versatile, multifunctional biomolecule. For example, riboswitches^1^ and ribozymes^2^ both frequently exhibit large-scale conformational fluctuations that underlie their gene regulatory and catalytic functions, respectively, and local conformational changes such as base flipping are important in recognition of RNA by certain proteins.^3^ Fluorescent base analogs (FBAs), which are chemically modified variants of the “native” bases A, C, G and U, enable local conformational changes at specific sites within RNA to be monitored spectroscopically. A large library of FBAs has been developed, offering analogues for every native base,^4–9^ including analogues suitable for intracellular microscopy^10,11^ and single-molecule detection.^12,13^ The adenine analogue 2-aminopurine (2AP) remains the most commonly used FBA for *in vitro* structural studies due to its broad commercial availability, and distinctive spectroscopic properties that enable assessment of the degree of base stacking and local polarity at the site of substitution.^14^

Recent investigations by the Widom lab highlighted two features of 2AP that have particularly notable implications for its use to study RNA structure: (1) That 2AP emission is severely quenched in base-stacked conformations,^15^ and (2) that replacement of adenine with 2AP can impact the structure and stability of functional RNAs by altering the Hoogsteen face of the base and by destabilizing its stacking with adjacent bases.^16^ Heterogeneity in base stacking of 2AP-labeled RNA was analyzed by fluorescence lifetime measurements^15,16^ and fluorescence-detected circular dichroism spectroscopy (FDCD),^15^ but analogous measurements can not be performed on unlabeled RNA, precluding direct inference of the impact of 2AP substitution on the stacking free energy landscape. These measurements yielded a general sense of 2AP′s degree of base stacking and solvent exposure in each spectroscopically distinguishable conformational state, but structures could be assigned only coarsely. Thus, an atomic-level understanding of the structures that give rise to the observed states remained lacking.

These limitations of experimental studies can be addressed using computational methods, such as all-atom molecular dynamics (MD)^17^ simulations, which can resolve atomistic structures of the different metastable states of RNA molecules.^18–22^ In addition, MD simulations can provide structural information for both fluorescent^23^ and non-fluorescent^24,25^ RNA molecules, enabling comparison of the conformational landscapes of FBA-substituted and unsubstituted RNA. However, MD simulation approaches suffer from two key limitations. First, non-polarizable classical force fields^18,26–29^ have limited accuracy. Moreover, for modified nucleobases, the force field parameters often need to be optimized by the user and are not as extensively tested for accuracy as those for standard RNA. Second, conformational transitions in RNA are rare events occurring on timescales of several microseconds to milliseconds,^30^ making it difficult to obtain ergodic sampling of the full conformational landscape at an affordable computational cost. To address the latter, several enhanced sampling methods^30–32^ have been introduced that apply an external biasing force to perturb the molecular potential energy surface and facilitate sampling of rare transitions across high barriers on a rugged free-energy landscape. Notable examples of such enhanced sampling methods include umbrella sampling,^33^ metadynamics,^34,35^ on-the-fly probability enhanced sampling (OPES),^36,37^ Gaussian accelerated molecular dynamics (GaMD),^38^ and Hamiltonian replica exchange,^39^ many of which have been applied extensively to study RNA conformational dynamics.^40,41^

These enhanced sampling methods often require identifying a low-dimensional order-parameter space, known as the collective variables (CV), along which the biasing force is applied to accelerate sampling. For conformational transitions in complex biomolecules like RNA, identifying a good CV capable of distinguishing among all relevant metastable states and saddle points is challenging. Machine learning algorithms, particularly deep neural networks (NN), have found considerable success in identifying suitable CVs for complex molecular processes,^42–45^ through either linear or non-linear combinations of molecular descriptors such as interatomic distances and dihedral angles. Such machine learning CVs have been used recently to study RNA conformational dynamics and ligand binding.^46–48^ However, NN CVs often have limited interpretability due to the black-box nature of deep learning models and incur high computational costs during the propagation of MD trajectories. The Ray group has recently introduced an explainable artificial-intelligence-based surrogate-model framework that approximates the neural-network CV as a linear combination of a subset of the original molecular descriptor set, leading to improved interpretability and reduced computational cost for enhanced-sampling simulations.^49^ Such methods have been benchmarked against long unbiased simulations to reproduce an accurate conformational landscape of RNA tetramers.^50^

**Fig. 1:**
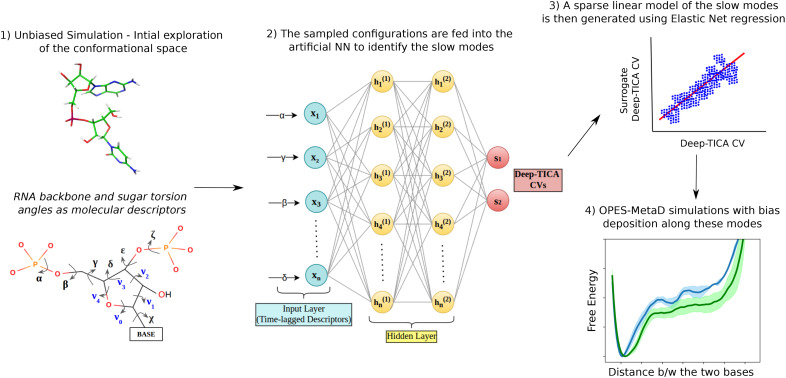
Schematic of the machine learning-driven collective variable discovery and enhanced sampling workflow used in this work to sample the conformational space of RNA dinucleotides and trinucleotides.

In this work, we perform enhanced-sampling simulations using our surrogate-model CVs to sample the base-stacking conformational landscape of 2AP-substituted and unsubstituted RNA dinucleotides and trinucleotides (Fig. 1) for which experimental fluorescence measurements were obtained in an earlier study.^15^ We report a comparative analysis of the free energy landscapes of different species and elucidate the structural ensembles of distinct stacking states to interpret experimental observations in atomistic detail.

## Computational methods

### System preparation and molecular dynamics simulation

In total, we simulated six dinucleotides (5′-A-A-3′, 5′-2AP–2AP-3′, 5′-A-C-3′, 5′-2AP-C-3′, 5′-C-A-3′, and 5′-C-2AP-3′) and two trinucleotides (5′-C-A-C-3′ and 5′-C-2AP-C-3′) to understand the role of 2AP substitution in RNA conformational landscapes. For the sake of simplicity, we drop the 5′ and 3′ notations for the rest of the manuscript. We constructed the initial structures of 2AP-substituted and unsubstituted RNA dinucleotides and trinucleotides using the tleap module of the AmberTools package. The 2AP base differs from its isomer, adenine, only because the NH_2_ group is located at the 2 position rather than the 6 position. Therefore, the force field parameters for the 2AP ribonucleotide were kept identical to those of adenine in the OL3 force field, except that the partial atomic charges and atom types were updated according to Remington *et al.*^23^ Although they studied a 2AP-containing DNA dinucleotide, they optimized the partial charges of a methyl-capped isolated 2AP base, making it possible to transfer the partial charges to an RNA system. The force field parameter files are provided at the GitHub link in the Data Availability section.

All MD simulations were performed in the CUDA-enabled version of GROMACS-2024.3,^51^ patched with PLUMED 2.11.^52^ All RNA systems were modeled using the AMBER OL3 force field,^53^ and the TIP3P^54^ water model was used to describe the solvent. Each system was solvated in a cubic box with a minimum water padding of 10 Å in all directions. Na^+^ and Cl^−^ ions were added to replicate the experimental^15^ ionic strength. All the solvated structures were prepared using the tleap program in the AmberTools suite and converted to the GROMACS format using the ParmEd package. Each system was energy-minimized using the steepest descent algorithm, followed by 2 ns of equilibration in the NVT ensemble and 1 µs of production in the NPT ensemble. The MD simulations were performed with a timestep of 2 fs. The long-range electrostatic calculation was carried out using the Particle Mesh Ewald (PME)^55^ summation. Temperature and pressure were maintained using the stochastic velocity rescale^56^ thermostat and the Parrinello–Rahman barostat,^57^ respectively.

### Training of machine learning collective variable

To perform enhanced-sampling simulations to exhaustively sample the conformational landscape, we designed a machine-learning collective variable using deep time-lagged independent component analysis (Deep-TICA)^58^ in combination with the Elastic-Net Regression^59^ surrogate model. Deep-TICA is a non-linear version of the original TICA algorithm.^60^ Deep-TICA employs a feedforward neural network to identify the slow modes as a function of molecular descriptors. This is acquired by the singular value decomposition of the time-lagged covariance matrix *C*(*τ*) constructed from the last hidden layer of the neural network.

1 *C*(*τ*)*α*_*i*_ = *λ̃*_*i*_*C*(0)*α*_*i*_

where *α*_*i*_ is the *i*-th eigenvector and the *λ̃*_*i*_ is the corresponding eigenvalue. The elements of the matrix *C*(*τ*) are given by

2 *C*_*ij*_(*τ*) = 〈*h*^*θ*^_*i*_ (*d*(*t*))*h*^*θ*^_*j*_(*d*(*t* + *τ*))〉

the hidden nodes *h*^*θ*^_*i*_ form the nonlinear combinations of the molecular descriptors set *d* at time *t* through the NN parameters *θ*. The lag time *τ* is chosen to filter out the fast dynamical modes that relax during the unbiased simulations, which are not necessary to include in the CV space for the enhanced sampling. Then we employed a surrogate-model approach based on Elastic-Net Regression^59^ to build an interpretable and computationally efficient CV, following our earlier work. The surrogate model approximates the neural network's input–output relationship as a sparse linear combination of the input descriptors, involving only the most relevant descriptors.3
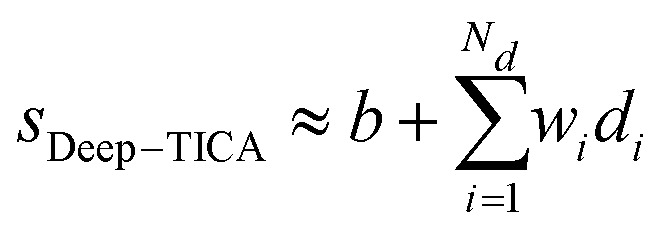


The weights (*w*_*i*_) and bias (*b*) are adjusted by minimizing the loss function 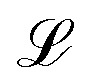, which is defined by,4
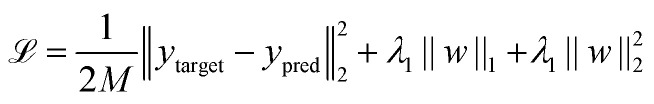
here, *M* is the number of time-lagged conformations in the input descriptor space, and *y*_target_, *y*_pred_, and *w* are vectors containing the target values (*i.e.*, *s*_Deep–TICA_), predicted values, and weights, respectively. The hyperparameters *λ*_1_ and *λ*_2_ are optimized using 5-fold cross-validation. We used the RNA backbone torsion angles^61^ (*α*, *β*, *γ*, *δ*, *ε*, and *ζ*), glycosidic angles (*χ*) and the sugar puckering angles (*v*_0_,*v*_1_,*v*_2_,*v*_3_, and *v*_4_) as the molecular descriptors for training the neural network and the surrogate model. The Deep-TICA and its corresponding surrogate model CV were trained from the 1 µs unbiased trajectory, which exhibited a limited number of unstacking events.

### Enhanced sampling simulations

To efficiently sample the conformational space of the oligonucleotides, we utilized the OPES,^36^ a variant of the popular method, WT-MetaD.^34,35^ Unlike the WT-MetaD, where the Gaussian hills are deposited along the CV space to construct the bias potential, in OPES, the Gaussian kernels are used to compute the marginal probability distribution *P*(*s*). The OPES method samples the well-tempered probability distribution *p*^tg^(*s*) ∝ [*P*(*s*)]^1/*γ*^, where *γ* is the bias factor that controls the smoothness of the distribution. The corresponding bias potential that yields the target distribution *p*^tg^(*s*) is given by5
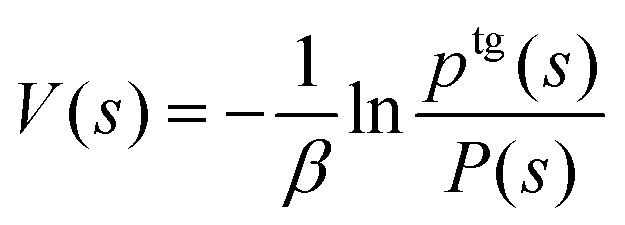


A regularization term *ε* and a normalization factor *Z* is incorporated to the bias potential equation. With these corrections, the bias potential *V*_*n*_(*s*) at the *n*th iteration of the OPES simulation is of the form,6
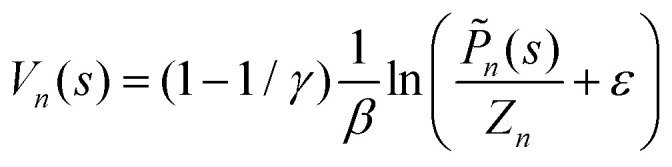
where *P̃*_*n*_(*s*) is the estimated marginal probability distribution along the CV space *s*. This estimate is given by:7
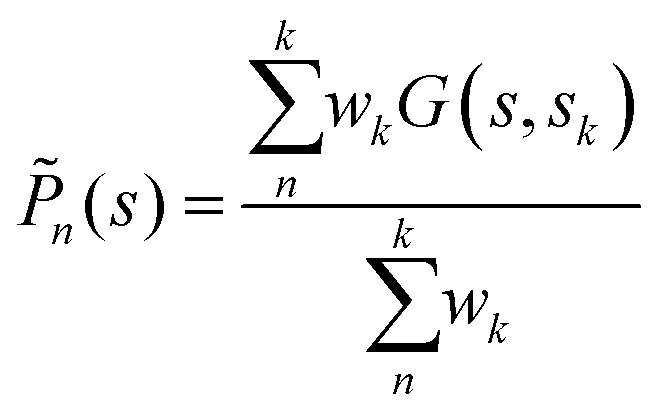
where *G*(*s*,*s*_*k*_) represents the Gaussian kernel centered at *s*_*k*_, and *w*_*k*_ is the weight associated with the *k*-th kernel, given by *w*_*k*_ = (*βV*_*k*−1_(*s*_*k*_)).

For all the systems, 3 replicas of 200 ns of the OPES simulations were performed by biasing the 2 slowest modes of the Surrogate-Deep-TICA CV. The BARRIER parameter and the bias deposition stride were set to 30 kJ mol^−1^ and 1 ps, respectively, and were kept identical for all the systems. The free energy surfaces are reconstructed by reweighting the biased trajectories. The unbiased marginal probability distribution, *P*(*s*), can then be obtained by ensemble averaging over the reweighted trajectory:8
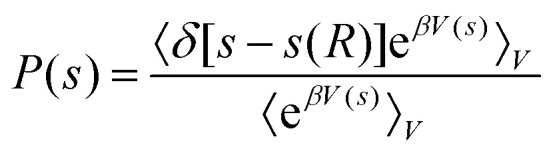
from which the unbiased free energy landscape is reconstructed:9
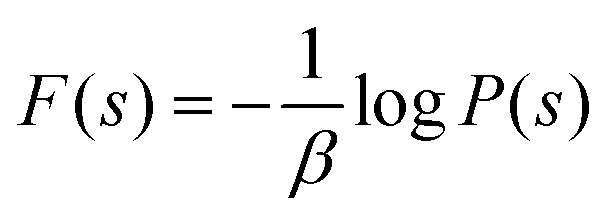


### Analysis of RNA conformational landscape

**Fig. 2:**
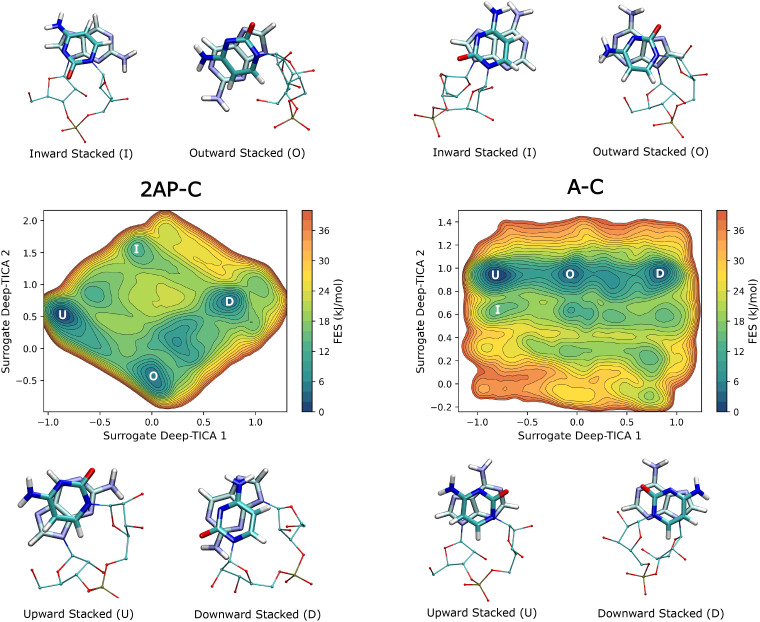
Free energy landscapes of 2AP-substituted and unsubstituted A–C dinucleotides projected along the two slowest modes of the surrogate deep-TICA CV. The free energy basins have been labeled according to the base stacking modes they represent. Representative structures for the different stacking modes are provided. The bases are shown in licorice representation, whereas the sugar phosphate backbone is shown in lines.

To study the stacking of different RNA oligonucleotides, we used the barnaba package developed by Bottaro *et al.*^62^ The structural criteria for stacking, as described in earlier work,^62,63^ are used. For dinucleotides, we also classified stacked structures into four stacking modes: Upward, Downward, Outward, and Inward (Fig. 2). Detailed structural description of these stacking modes can be found elsewhere.^64^

We quantified the populations of stacked and unstacked states, as well as different stacking modes, from the quasistatic bias regime of the reweighted OPES trajectories, after discarding the first 25% (*i.e.*, 50 ns) of each OPES run. Then, for dinucleotides, we classify each frame into one of five categories: unstacked, upward stacked, downward stacked, inward stacked, and outward stacked. In the case of trinucleotides, we also use five categories: all three bases unstacked, all three bases stacked, only the first two bases stacked, only the last two bases stacked, only the first and third bases stacked, and one of the terminal bases intercalated between the other two. The population *p*_*k*_ of the *k*-th category (*C*_*k*_) is computed as:
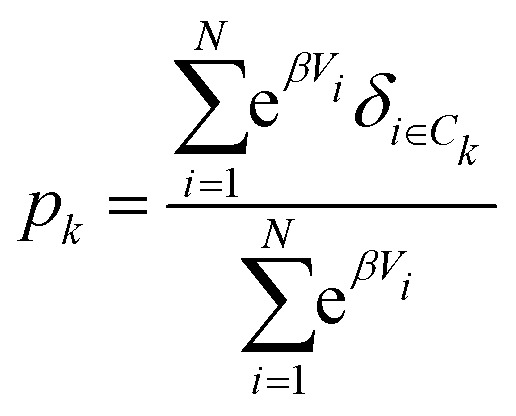
where *β* = 1/*k*_B_*T*, *V*_*i*_ is the OPES bias on the *i*-th trajectory frame, *N* is the total number of frames analyzed, and *δ*_*i*∈*C*_*k*__ = 1 if *i*-th frame falls within class *C*_*k*_ and zero otherwise. The population for each stacking category is calculated from three independent OPES runs, and the mean and uncertainties are reported. To calculate the population of the stacked conformations for the dinucleotides, we summed over the populations of all four stacking modes. In the case of the trinucleotides, the population of the state we refer to as “adenine-stacked” is computed by summing over the populations of those configurations where the adenine or the 2AP base is stacked with any neighboring base.

## Results

### Conformational diversity and slow modes of RNA dinucleotides

Our enhanced sampling simulations revealed a rich conformational diversity in both 2AP-substituted and unsubstituted RNA dinucleotides. The projection of the free energy landscape onto the two Surrogate Deep-TICA modes does not necessarily follow simple stacking-unstacking dynamics; rather, it captures subtle conformational changes among different stacking modes in the system. The free energy landscapes reveal several deep and shallow free-energy minima, each corresponding to a different base-stacking state of the system (Fig. 2). For example, in the case of the C-2AP dinucleotide, we observe that the two deepest minima correspond to the upward- and downward-stacked conformations, whereas the inward- and outward-stacked conformations are less populated. A rugged and complex free energy landscape is also observed for the unmodified C-A dinucleotide, where all three deepest minima are distinguished by the first slowest mode, *i.e.*, surrogate-Deep-TICA 1, unlike the C-2AP system, where both Deep-TICA components were required to distinguish between the different stacking modes (Fig. 2). This demonstrates that even when the 2AP substitution does not significantly change the relative populations of stacked and unstacked states, it can affect the system's overall dynamics and slow modes (as seen in the difference between the FES projections along the two slowest modes) and shift population among different stacking modes.

**Fig. 3:**
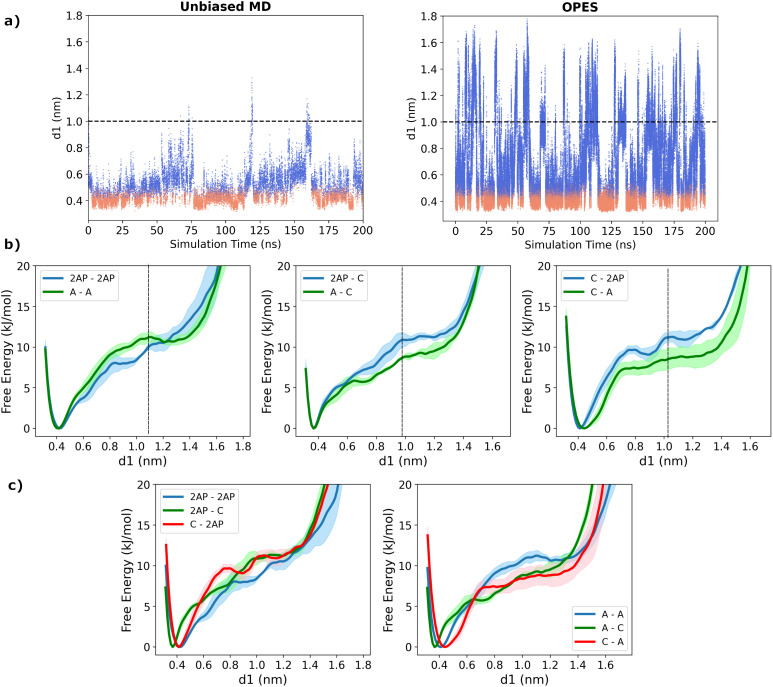
(a) Evolution of the distance (*d*1) between the six-membered rings of the two 2AP bases of the 2AP–2AP dinucleotide as a function of simulation time for OPES and unbiased simulation. The black dashed line indicates the location of the highest free energy barrier along (*d*1). The structures categorized into stacked conformation using barnaba software are marked in orange, while others are marked in blue. Only the first 200 ns of the unbiased trajectory is shown for a direct visual comparison of the frequency of transitions across the barrier. Similar results for other systems are provided in SI (Fig. S2 and S4). (b) One-dimensional projections of the free energy landscapes of the RNA dinucleotides along *d*1. Comparison is shown between 2AP-substituted and unsubstituted dinucleotides with the same sequence. (c) Same as (b), but comparison is shown between different sequences for 2AP-substituted and unsubstituted dinucleotides. All uncertainties were computed from three independent OPES simulations.

The inability of the surrogate-Deep-TICA CV to capture the stacking-unstacking process specifically can be attributed to the fact that the unstacked conformation does not correspond to a single deep free-energy minimum, and consequently, such transitions may not be the slowest modes of the system. Nevertheless, the OPES simulations could sample several back-and-forth transitions between the stacked and unstacked conformations, indicating that the trained CVs do accelerate sampling of the base-stacking process (Fig. 3a and SI Fig. S2 and S4). To investigate this further, we analyzed the feature weights of the Surrogate Deep-TICA CVs. The most important features in terms of the absolute values of the coefficients of the Elastic Net Regression models often involve the *γ*, *β*, and *χ* torsion angles, which are directly responsible for distinguishing between different stacking conformations (SI Fig. S5–S12). In contrast, the sugar puckering angles receive much lower importance in the CV, indicating that transitions between sugar puckering forms are not the slowest or most relevant dynamical processes in these systems. Despite providing all possible torsion angles during training, our surrogate model can automatically select the relevant torsion angles for the process at hand, demonstrating a strong ability to automate feature selection.

### Effect of 2AP substitution in modulating the dinucleotide conformational landscape

As the distance (*d*1) between the centers of the six-membered rings of the two bases can distinguish between the stacked and unstacked conformations more intuitively than the torsion angles, we project the free energy landscape along the *d*1 collective variable for further analysis. It should be noted, however, that ML CVs trained on dihedral-angle descriptors provide a more detailed description of the stacking modes within the stacked ensemble and enable extensive sampling of the conformational landscape.

**Fig. 4:**
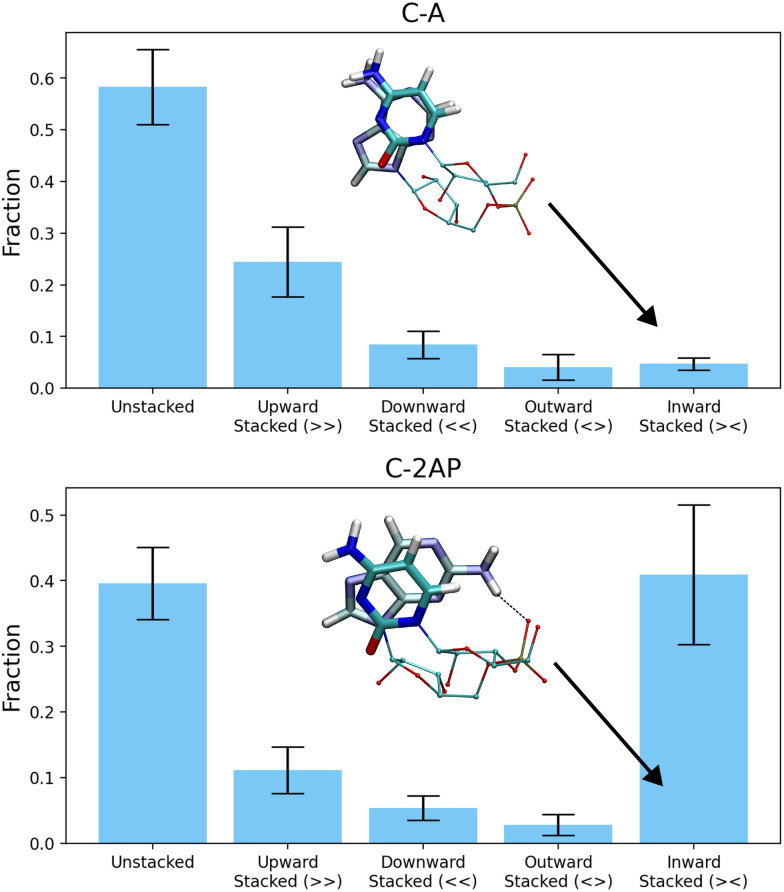
Normalized population of different stacking modes present in C-A and C-2AP dinucleotides. Representative structures are shown for the inward stacked ensemble, which exhibits the largest change in abundance due to 2AP substitution.

Notably, the 2AP substitution does not significantly alter the one-dimensional free-energy profiles along *d*1 (Fig. 3). All systems indicate a single deep free-energy minimum corresponding to the stacked conformations, and higher free-energy plateaus representing unstacked states. Each system shows the highest free-energy barrier at around *d*1 ∼ 1.0 nm. Note that although the unbiased MD simulations do sample excursions away from the stacked conformation, they struggle to escape the deep free energy basin, and the barrier around *d*1 ∼ 1.0 nm could be crossed consistently only in the OPES simulations (Fig. 3a). Therefore, even for seemingly simple systems such as RNA dinucleotides, enhanced-sampling simulations may be required to adequately explore the conformational space. 2AP substitution only slightly increases the relative stability of stacking with the C base, in both 5′ and 3′ directions. Contrarily, the 2AP–2AP dinucleotide exhibits a slightly destabilized stacked state compared to the A-A dinucleotide. These results are consistent with the fractions of stacked and unstacked conformations computed using the stricter criteria implemented in the barnaba software (SI Fig. S13). The only qualitative difference that is observed in the free energy profiles of 2AP-substituted and unsubstituted dinucleotides is that the basin corresponding to the stacked state in the C-A dinucleotide is broader than that of the C-2AP. The unstacked state of C-A also exhibits greater stabilization than that of C-2AP, resulting in a significant increase in the population of the stacked conformation (1.5 times compared to that in the unmodified dinucleotide) (Fig. 4). In contrast, the change in the population of the stacked state is relatively modest for other studied dinucleotides. This discrepancy arises from the greater population of inward-stacked conformations in the C-2AP system compared to the C-A system (Fig. 4). In the case of C-A dinucleotide, the amine groups of the two bases remain in an eclipsed conformation in the inward stacked conformation, leading to electrostatic repulsion that reduces the stability of the conformation. Contrarily, in the inward stacked conformation of the C-2AP system, the amine groups of the two bases are 120° apart from each other, avoiding electrostatic repulsion (Fig. 4). It also allows for the amine group of 2AP to form a hydrogen bond with the backbone phosphate group, further stabilizing the inward stacked conformation.

### Base stacking heterogeneity in RNA trinucleotides

**Fig. 5:**
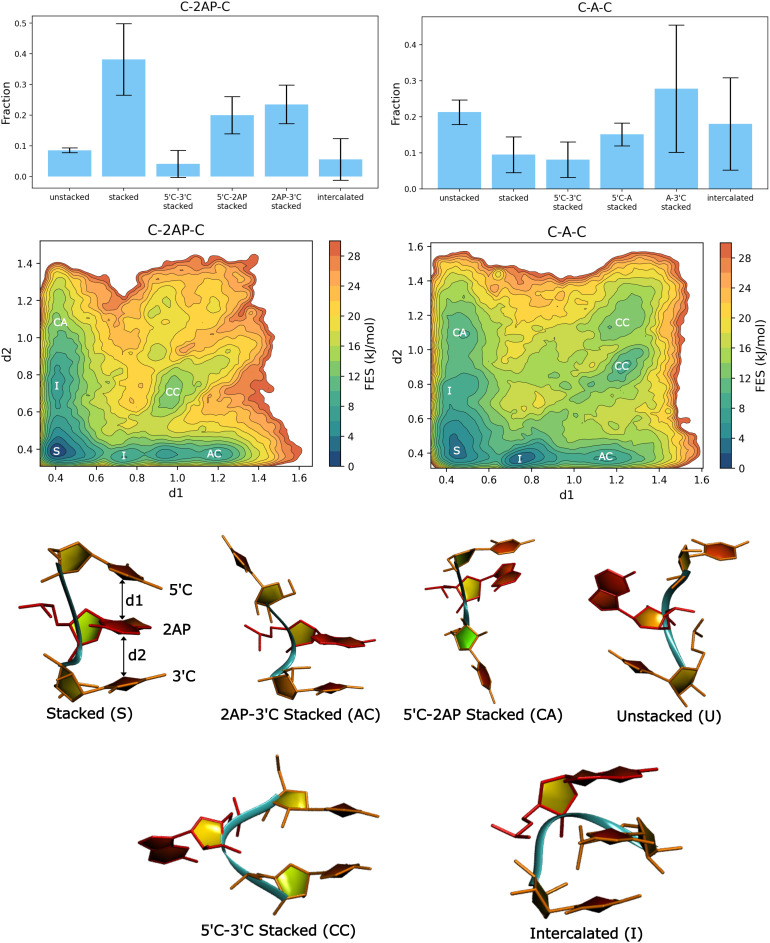
Comparison between 2AP-substituted and unsubstituted C-A-C trinucleotides. (Top panel) The relative populations of the different stacking states for the two systems. (Middle panel) The free-energy landscapes for the two systems, projected onto the *d*1 and *d*2 CVs. The free energy basins are labeled with the stacking modes they represent. (Bottom panel) Representative structures of the different stacking modes. The different bases are labeled for the fully stacked conformation, and the two distance-based CVs used for projecting the FES are depicted. Representative structures are shown here only for the C-2AP-C system, and analogous representations for the unsubstituted trinucleotide are provided in the SI.

In RNA trinucleotide systems, we observed complex free-energy landscapes along the Surrogate Deep-TICA modes, similar to those in dinucleotide systems (SI Fig. S20). From visual inspection, we classified the conformations into six categories: (i) unstacked: none of the bases are stacked, (ii) stacked: all three bases are stacked. (iii) 5′C-3′C stacked and the A or 2AP is unstacked, (iv) 5′C-A or 5′C-2AP stacked and the 3′C is unstacked, (v) A-3′C or 2AP-3′C is stacked, and the 5′C is unstacked, (vi) intercalated: one C base intercalated between C and A (or 2AP) (Fig. 5 and SI Fig. S19). For better intuition, we projected the free energy landscape onto a two-dimensional space defined by the distances between the six-membered rings of consecutive bases, *d*1 and *d*2 (Fig. 5). In the 2AP substituted trinucleotide, the fully stacked conformation is most populated and corresponds to the deepest free energy minima around *d*1 ≈ *d*2 ≈ 0.4 nm. In the unmodified CAC trinucleotide, however, the intercalated conformation with a stacking between the A and 3′C is more prevalent (minima at *d*1 ≈ 0.7 nm and *d*2 ≈ 0.4 nm), where the 5'C is stacked with the 3′C instead of A. The overall abundance of fully unstacked and intercalated structures is also higher in the C-A-C trinucleotide than in its 2AP-tagged counterpart. These results are consistent with the observation in the dinucleotide, where the stacking tendency between C-A was consistently weaker than the 2AP-substituted systems, which, in the case of trinucleotide, facilitates the formation of intercalated and fully unstacked states.

### Correlation between base stacking and experimental dark state population

**Fig. 6:**
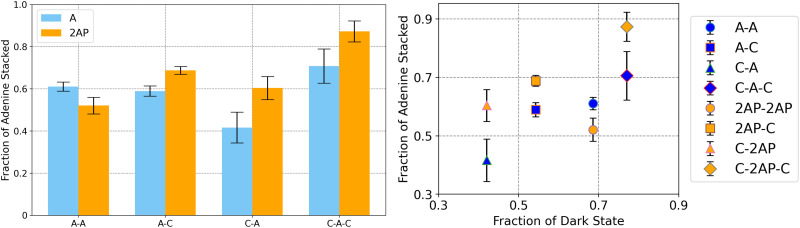
(Left) Comparison of the fraction of adenine stacked conformations obtained from OPES simulations for the dinucleotide and trinucleotide systems with and without the 2AP substitution. (Right) The fraction of adenine stacked conformations obtained from OPES simulations, plotted against the experimental dark-state populations from ref. 15.

One of the primary objectives of this work is to interpret the experimental fluorescence data in terms of the atomistic configurations of the oligonucleotides. When the 2AP is stacked with another base, its fluorescence is quenched, sometimes so severely that the stacked conformation appears completely dark in standard fluorescence measurements.^15^ Conversely, 2AP is highly emissive in unstacked conformations. Therefore, to interpret the experimental fluorescence data, we plotted the fraction of “adenine-stacked” states (*i.e.*, where the adenine or 2AP is stacked with a neighboring base, including intercalated structures in the trinucleotide) against the experimental dark state populations reported from fluorescence lifetime measurements in ref. 15 (Fig. 6). It should be noted here that adenine is non-emissive; therefore, fluorescence-based estimates of base-stacked populations are available only for the 2AP-modified oligonucleotides. We observe a clear positive correlation between the dark-state population and the fraction of adenine-stacked structures, indicating that 2AP stacking is the primary driver of dark-state formation. This trend also validates our simulation results by demonstrating consistency with experiments. The only exception to this trend is the 2AP–2AP dinucleotide, which shows a significantly lower population of stacked structures relative to its experimental dark-state population. This could be attributed to artifacts in the force-field parameters of 2AP, which may amplify errors when two such modified bases are in close proximity, causing certain states to be artificially over- or underpopulated. Furthermore, a stacked structure of 2AP–2AP represents a classic “H-type” exciton coupled homodimer.^65^ A face-to-face arrangement within a homodimer imparts quenching beyond that observed for 2AP when stacked with native bases, potentially leading to an overestimation of the dark-state population. The most notable observation is that the fraction of stacked conformations in the unmodified oligonucleotides is in excellent agreement with the experimental dark-state population of the 2AP-modified oligonucleotides. It demonstrates that the experimental results obtained with the 2AP-substituted dinucleotide and trinucleotides studied in this work are representative of the unmodified RNA oligonucleotides with identical sequence.

## Discussions and conclusion

In this work, we performed enhanced-sampling simulations of RNA dinucleotides and trinucleotides using machine-learning collective variables to determine how FBA substitutions affect their conformational landscapes. We observe no qualitative difference in the base stacking thermodynamics between 2AP-substituted and unsubstituted RNA systems, although the fraction of stacked structures may differ by ∼5–10%. A larger difference was observed only in the case of 5′C-A-3′, where 2AP tagging increased the population of the stacked structures by ∼20% as it stabilizes the inward stacked subpopulation by removing an energetically unfavorable NH_2_–NH_2_ eclipsed conformation and by forming a hydrogen bond between the NH_2_ group at the 2 position and an oxygen from the backbone phosphate group. This effect is also translated to the trinucleotide, where the stacking propensity between the 5′C and its neighboring A base is increased substantially by 2AP substitution, making the fully stacked structure the global free energy minimum, instead of the intercalated one, as is the case for the untagged CAC trinucleotide. All these results should, however, be interpreted in the context of the limited accuracy of the fixed-charge classical force field for RNA, which allows only qualitative comparisons between systems. In light of this caveat, we conclude that the effect of 2AP substitution on the base-stacking conformational landscape of small RNA oligonucleotides is minimal.

Notably, the Surrogate Deep-TICA collective variables, which are designed to identify the system's slow modes, do not distinguish well between stacked and unstacked conformations, possibly because the unstacked conformation is not a stable free-energy minimum. But they can discriminate well among the different stacking modes, as these structures constitute distinct minima on a rugged free energy landscape. Nevertheless, biasing along the Surrogate Deep-TICA CVs with the OPES algorithm yields several transitions between the stacked and unstacked states, enabling the calculation of converged and reproducible free-energy profiles.

Above all, our computational results are in direct agreement with the experimental work, as the population of adenine stacked conformations correlates with the population of the dark states observed in fluorescence spectroscopy experiments. This is consistent with the understanding that stacking quenches 2AP fluorescence, thereby forming dark states. Our work, however, does not directly predict the populations of individual bright states,^15^ as classical force-field models can only describe conformational dynamics and cannot capture the photophysics of modified nucleobases. Nevertheless, our results demonstrate that substituting adenine with 2AP may modulate the detailed atomistic free-energy landscape of these oligonucleotides without significantly altering the propensity for base stacking. However, determining whether these conclusions can be generalized to more complex, physiologically relevant RNA systems requires further investigation, which we aim to conduct in future follow-up studies.

## Author contributions

D. R. and J. R. W. designed and supervised the research; R. E. and E. S. performed and analyzed the simulations; D. R. and J. R. W. contributed to the analysis of the results. R. E., J. R. W., and D. R. wrote the paper.

## Conflicts of interest

The authors declare no competing financial interest.

## Supplementary Material

CP-028-D6CP01607C-s001

## Data Availability

The simulation input files and analysis scripts are provided in the GitHub repository: https://github.com/Revanth27-08/2-Aminopurine-Substituted-RNA.git. Supplementary information (SI): Additional results are provided in Fig. S1–S20 of the SI. See DOI: https://doi.org/10.1039/d6cp01607c.
