# Supplementary material for: Conformational landscape of 2-aminopurine-substituted RNA oligonucleotides from machine-learning-driven enhanced sampling
Source: Phys Chem Chem Phys. 2026 Jul 10;28(29):18062–71. doi: 10.1039/d6cp01607c (PMC13352520; doi:10.1039/d6cp01607c)
Supplement: CP-028-D6CP01607C-s001 [file CP-028-D6CP01607C-s001.pdf]

Supplementary Information (SI) for Physical Chemistry Chemical Physics.
This journal is © the Owner Societies 2026

Supporting Information: Conformational Landscape of 2-Aminopurine-Substituted RNA Oligonucleotides from Machine-Learning-Driven Enhanced Sampling

Revanth Elangovan,[†] Emily Stetson,[†] Julia R. Widom,[*,†,‡,¶] and Dhiman Ray[*,†,§,‖]

†*Department of Chemistry and Biochemistry, University of Oregon, Eugene, Oregon 97403, USA*

‡*Oregon Center for Optical, Molecular, and Quantum Science, University of Oregon, Eugene, Oregon 97403, USA*

¶*Institute of Molecular Biology, University of Oregon, Eugene, Oregon 97403, USA*

§*Materials Science Institute, University of Oregon, Eugene, Oregon 97403, USA*

‖*Department of Physics, University of Oregon, Eugene, OR 97403*

E-mail: jwidom@uoregon.edu; dray@uoregon.edu

1 Supplementary Methods

We employed the Deep-TICA[S1] algorithm in combination with an Elastic Net regression[S2] surrogate model for training our CV, the training was performed using the `mlcolvar` package[S3] together with `scikit-learn`.[S4] For the neural network architecture, we used a [40-24-12-5] topology for dinucleotides and [64-45-20-5] for trinucleotides, a lag time of 10 ps was employed, and the shifted softplus function was used as the activation function for the training. Structural ensembles corresponding to stacked, unstacked, and intercalated conformations, along with stacking analyses, were obtained using the `barnaba` toolkit.[S5] All other analyses were carried out using standard tools available in PLUMED[S6] and GROMACS.[S7] All the trajectories were visualized and analyzed using the VMD[S8] software.

1.1 Stacking Criteria

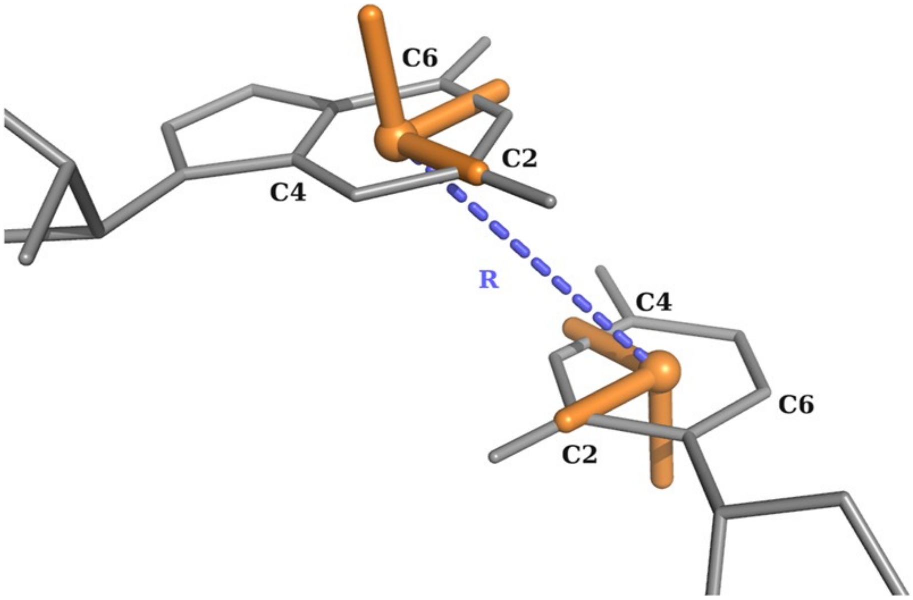

Figure S1: Definition of the local coordinate systems and of the vector R for purines and pyrimidines. Reprinted from Bottaro, S., *et al.* RNA, 25(2), 219-231.[S5] Copyright 2019 Bottaro et al.

The relative position and orientation of nucleobases were characterized using the `barnaba` toolkit. Stacking interactions were identified according to the criteria introduced in the original `barnaba` toolkit. In this approach, a local coordinate system is defined for each nucleotide using the C2, C4, and C6 atoms of the nucleobase, with the x-axis directed toward C2 and the y-axis directed toward C4 (C/U) or C6 (A/G). The position of nucleobase j in the reference frame of nucleobase i is represented by $\mathbf{R}_{ij} = (x_{ij},\ y_{ij},\ z_{ij})$, note that $|R_{ij}| = |R_{ji}|$ but $R_{ij} \neq R_{ji}$, the R_{ji} is the central definition of the eRMSD metric and annotation strategy. The stacking geometries are classified based on the sign of the z-coordinates

- upward (>>) if $z_{ij} > 0$ and $z_{ji} < 0$

- downward $(<<)$ if $z_{ij} < 0$ and $z_{ji} > 0$
- outward $(<>)$ if $z_{ij} < 0$ and $z_{ji} < 0$
- inward $(><)$ if $z_{ij} > 0$ and $z_{ji} > 0$

2 Supplementary Results

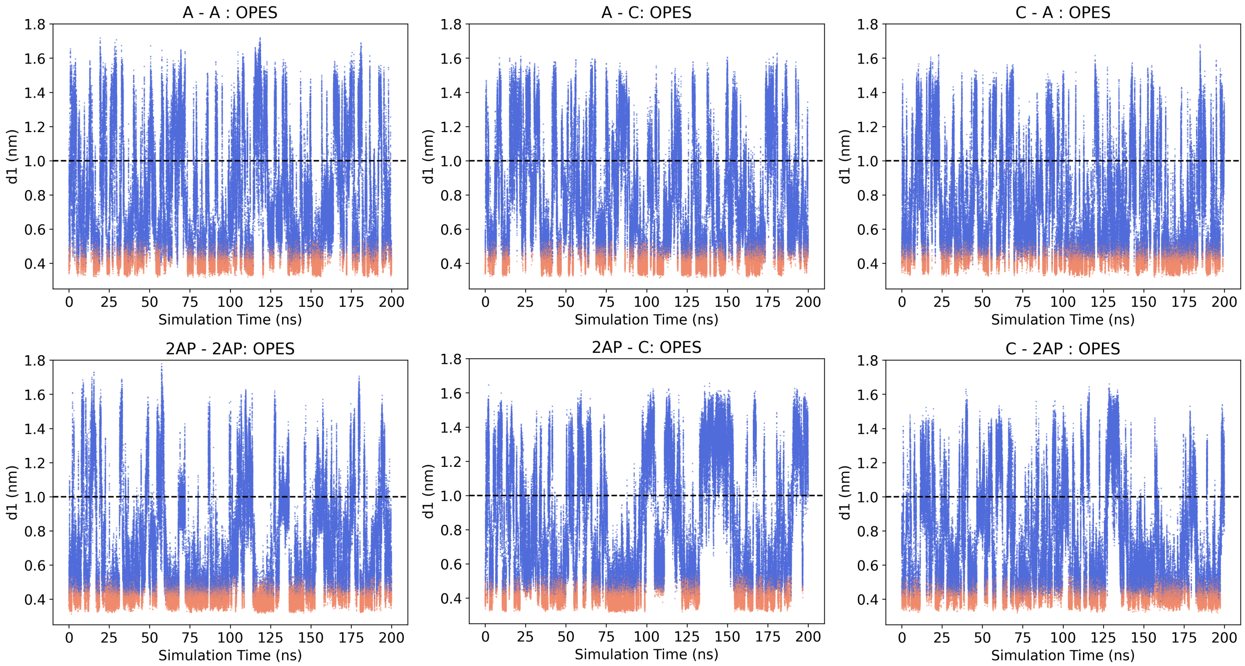

Figure S2: Evolution of the distance ($d1$) between the six-membered ring of the two bases of the 2AP-unsubstituted dinucleotide (upper panel) and the 2AP-substituted dinucleotide (bottom panel) as a function of simulation time for OPES. The black dashed line indicates the location of the highest free energy barrier along ($d1$). The structures categorized into stacked conformation using `barnaba` software are marked in orange, while others are marked in blue.

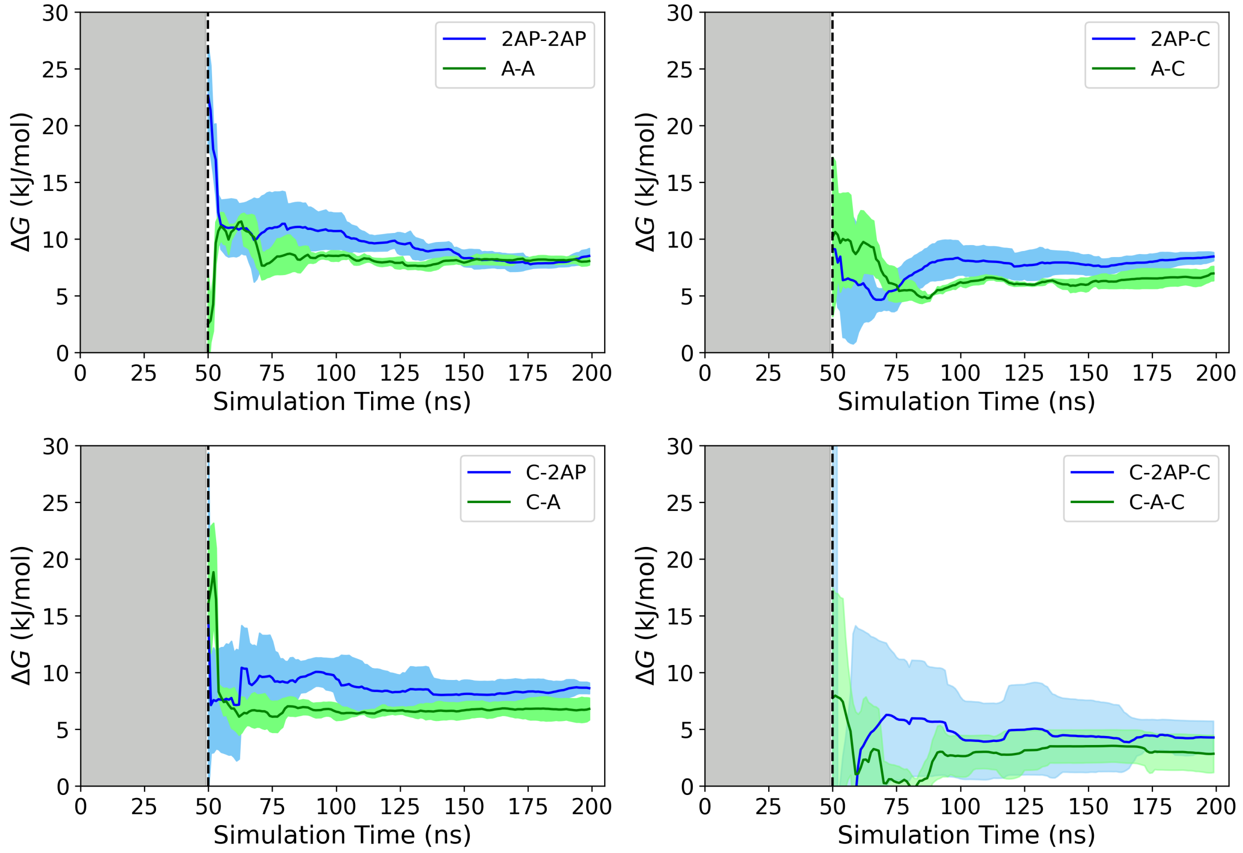

Figure S3: Convergence of the free energies from OPES simulations, the blue-shaded region (2AP-substituted) and green-shaded region (2AP-unsubstituted) depict a 95% confidence interval from three independent runs. The initial 50 ns shown in gray corresponds to the equilibration phase.

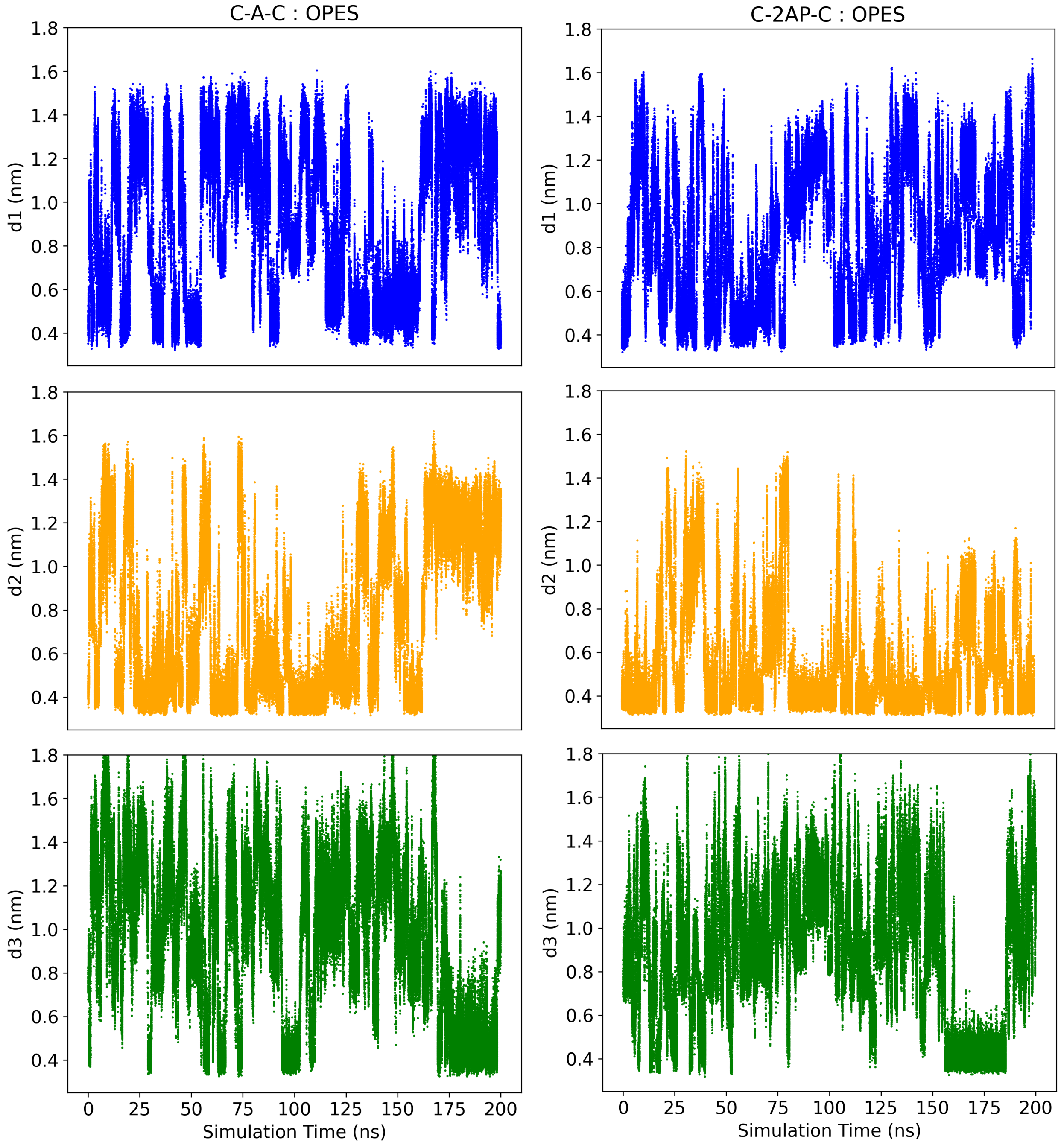

Figure S4: Evolution of the distances ($d1$ - distance between the six-membered ring of the $5'$C and A, $d2$ - distance between the six-membered ring of the A and $3'$C, $d3$ - distance between the six-membered ring of the $5'$C and $3'$C) as a function of simulation time of the 2AP-unsubstituted (left panel) and 2AP-substituted (right panel) for the OPES.

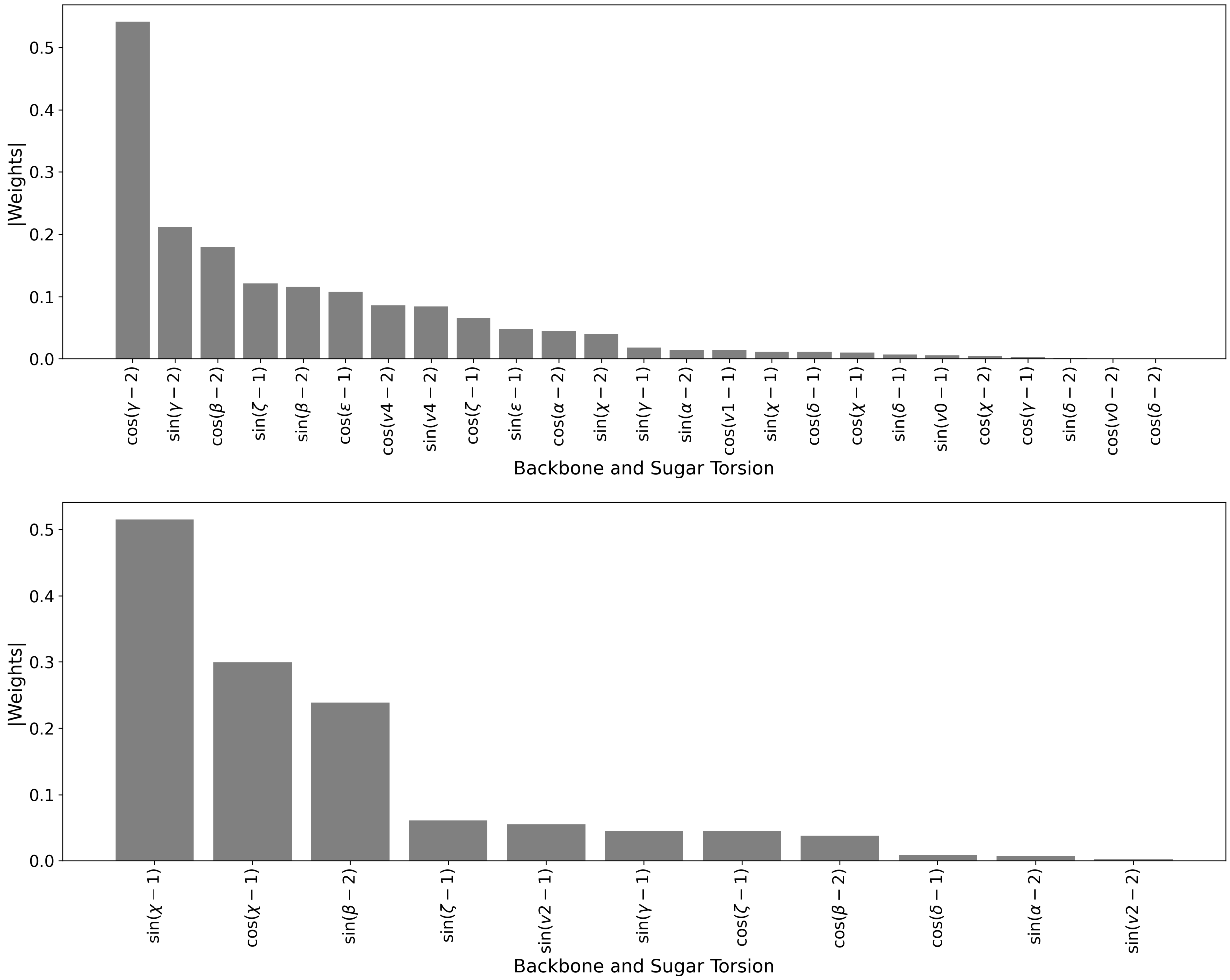

Figure S5: Magnitudes of the regression coefficients corresponding to the torsion descriptors with non-zero contribution in the surrogate models mode 1 (upper panel) and mode 2 (bottom panel) of the Deep-TICA CVs for the 2AP substituted A-A dimer.

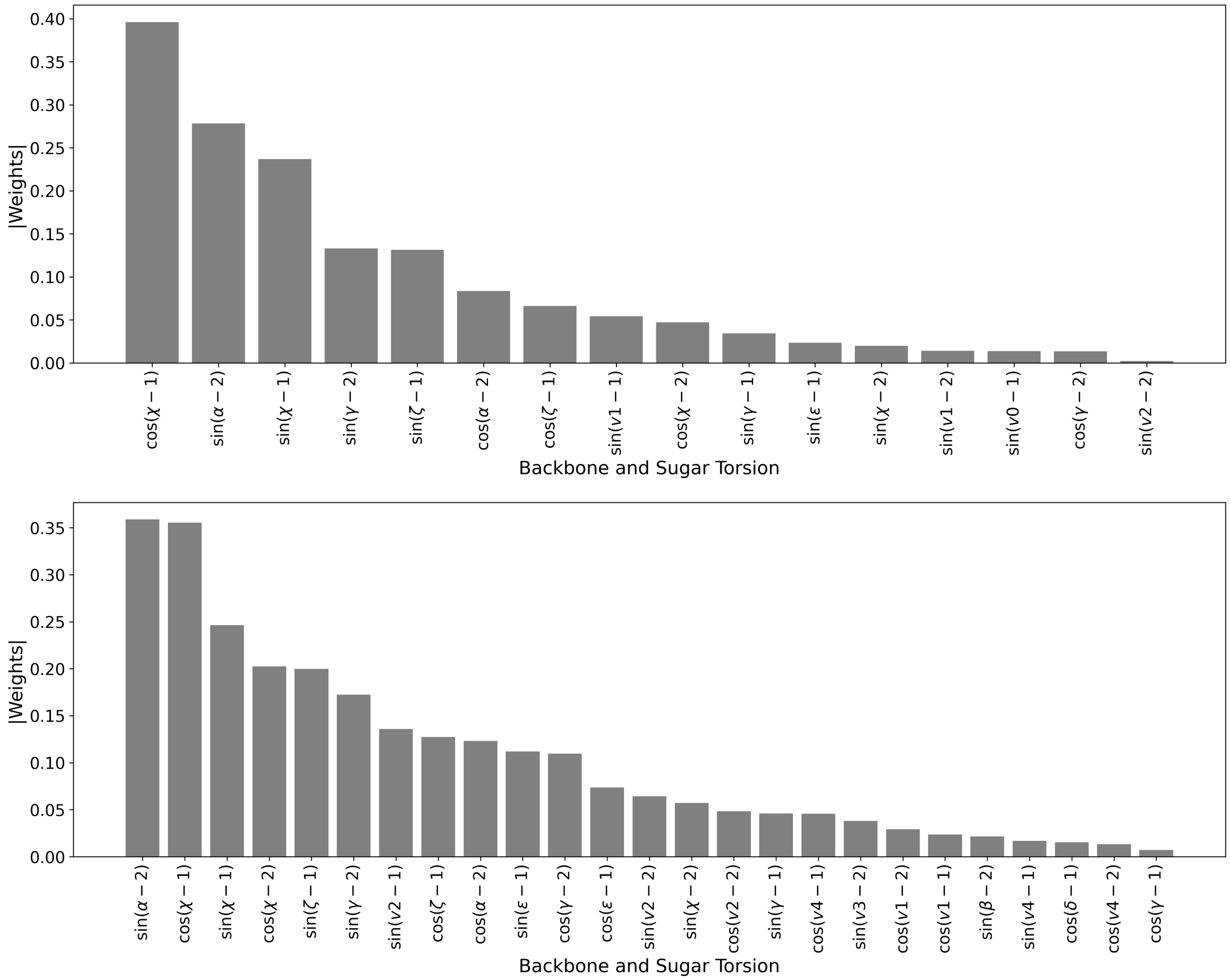

Figure S6: Same as Fig. S5, but for the 2AP substituted A-C dimer.

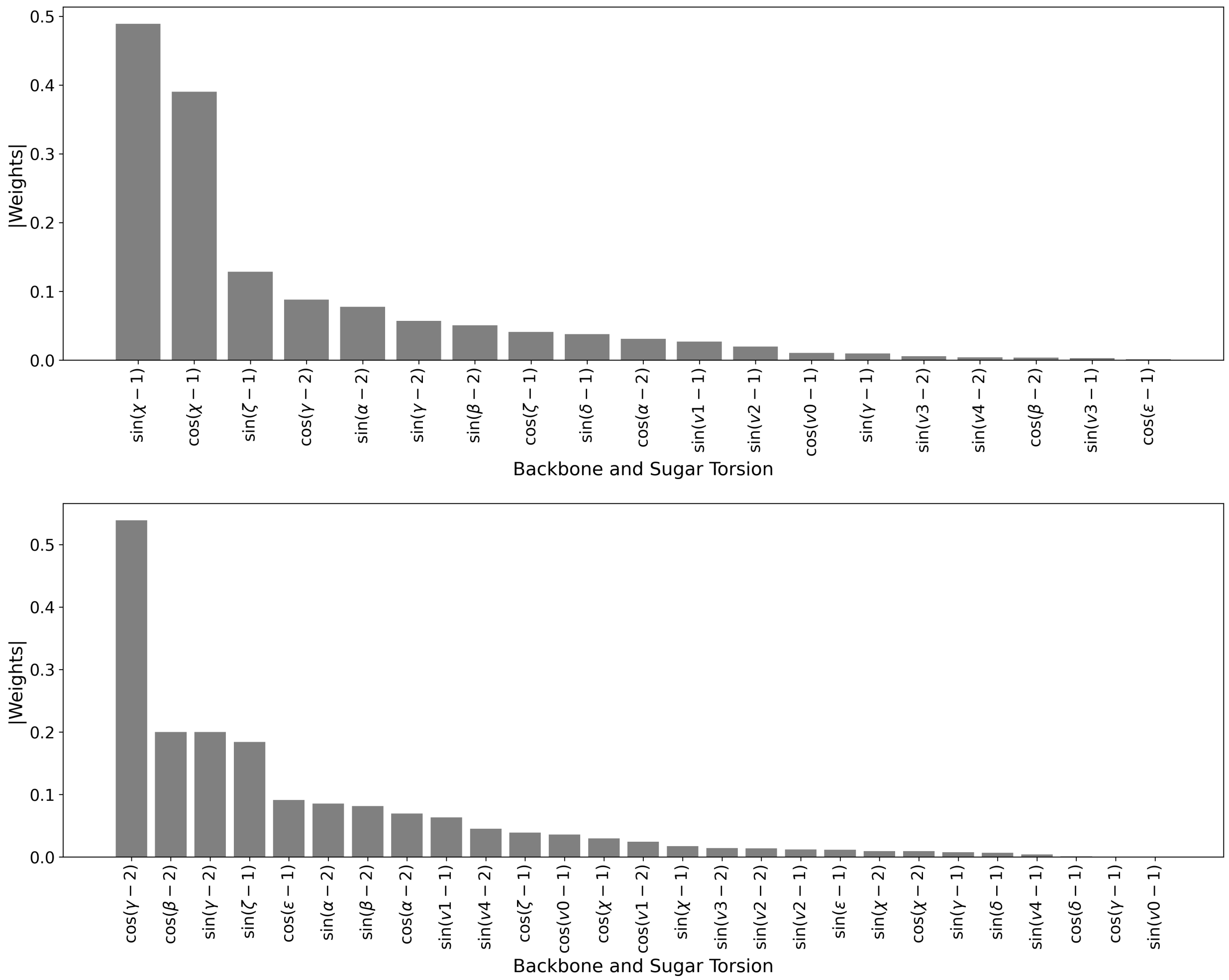

Figure S7: Same as Fig. S5, but for the 2AP substituted C-A dimer.

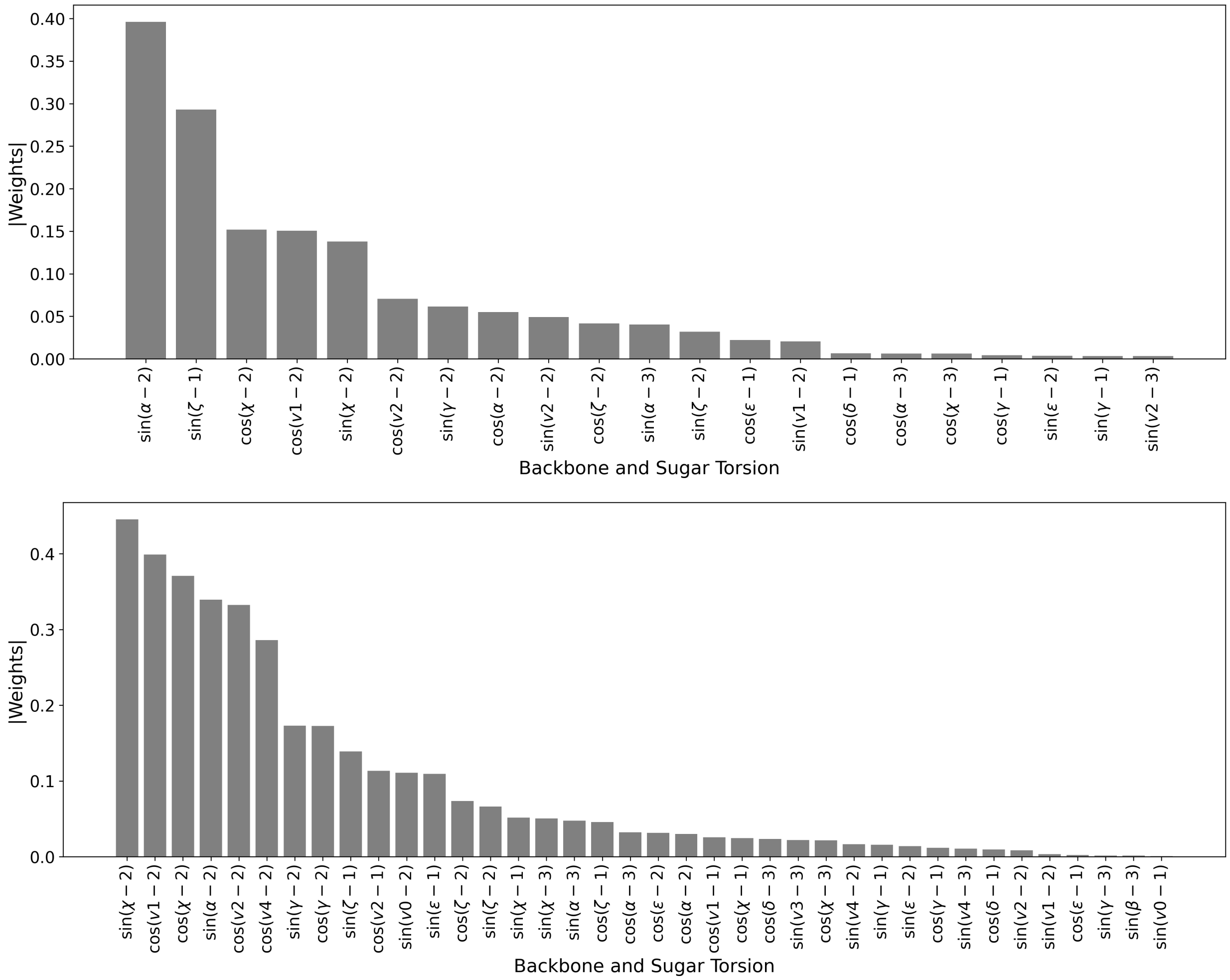

Figure S8: Same as Fig. S5, but for the 2AP substituted C-A-C trimer.

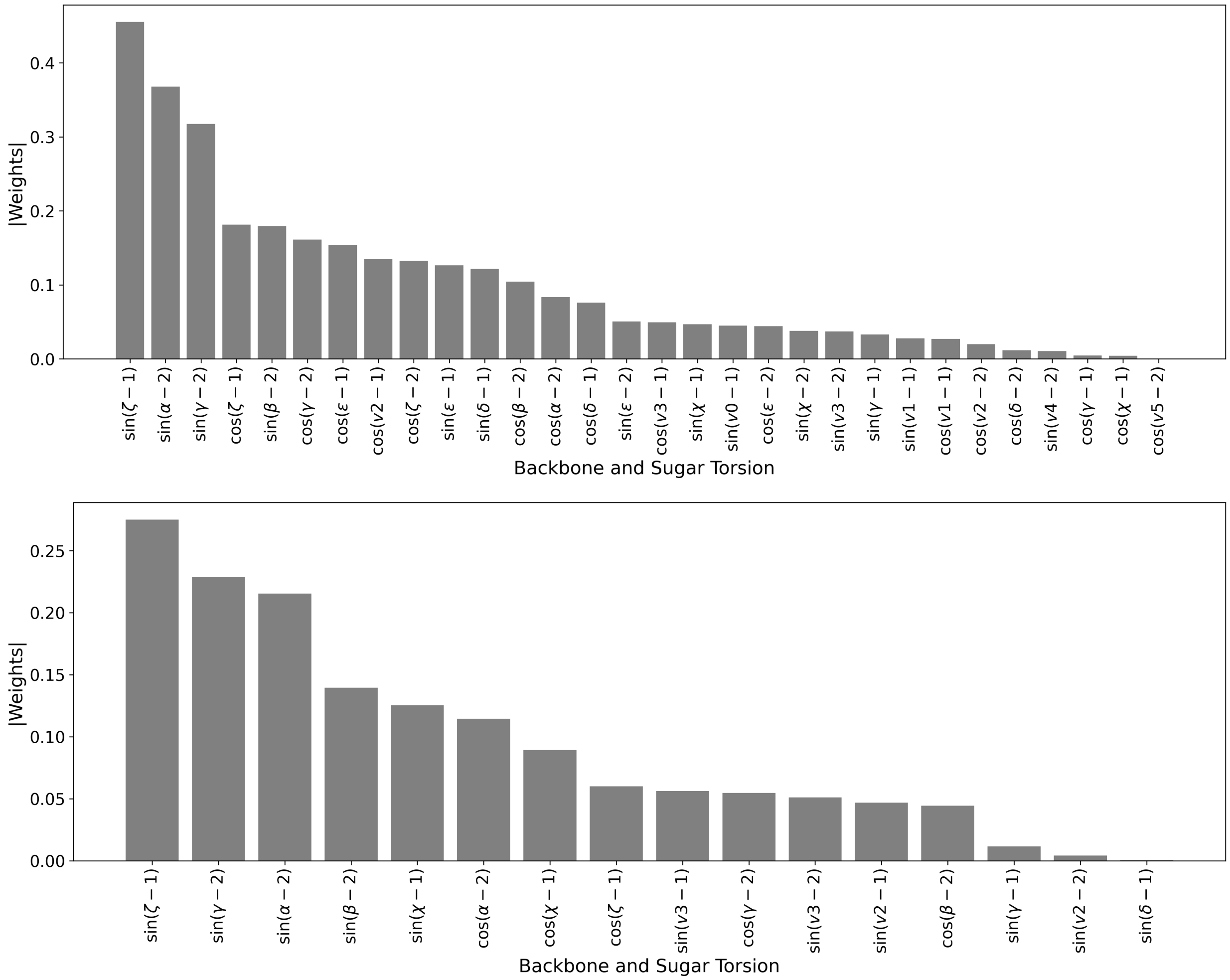

Figure S9: Same as Fig. S5, but for the 2AP unsubstituted A-A dimer.

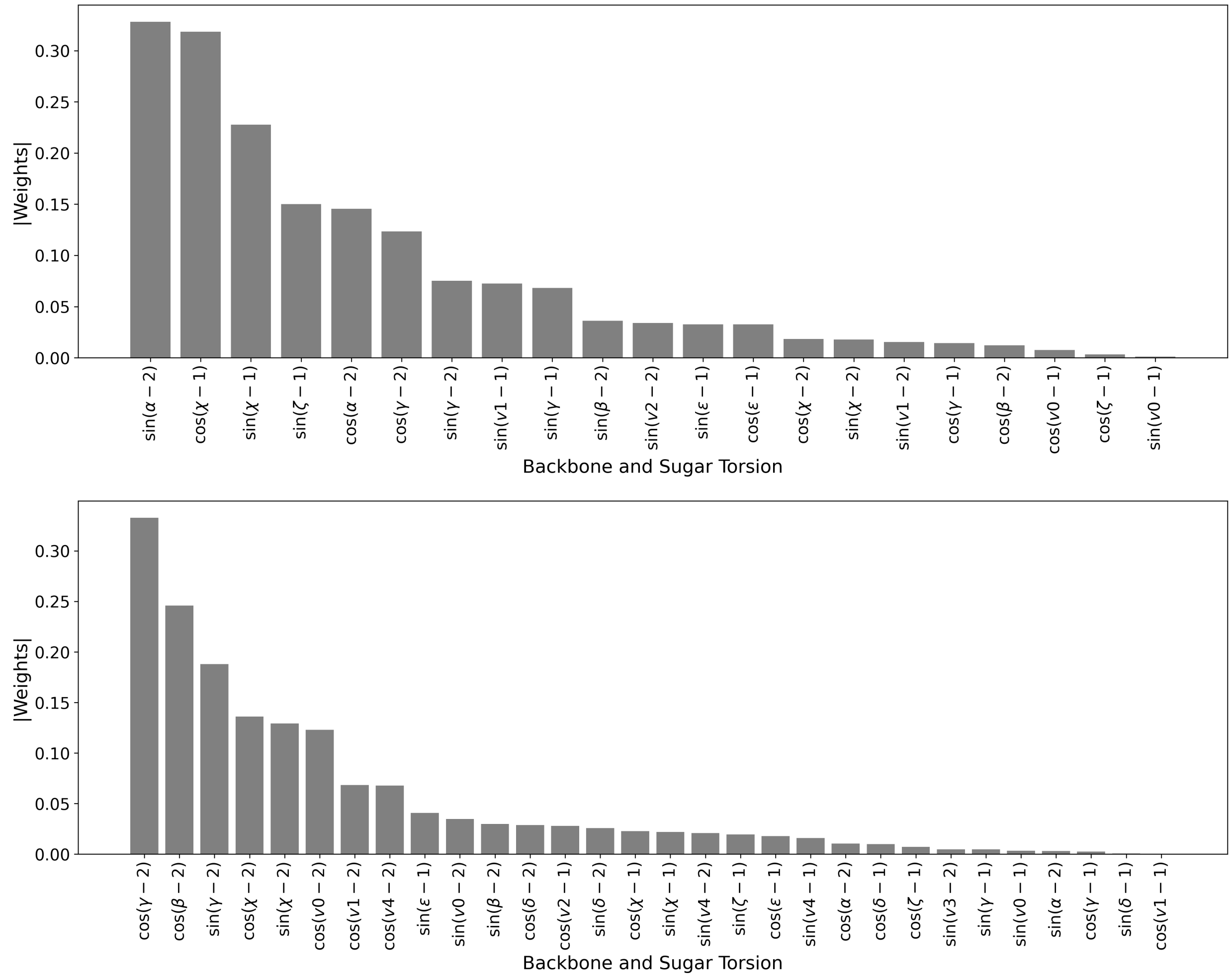

Figure S10: Same as Fig. S5, but for the 2AP unsubstituted A-C dimer.

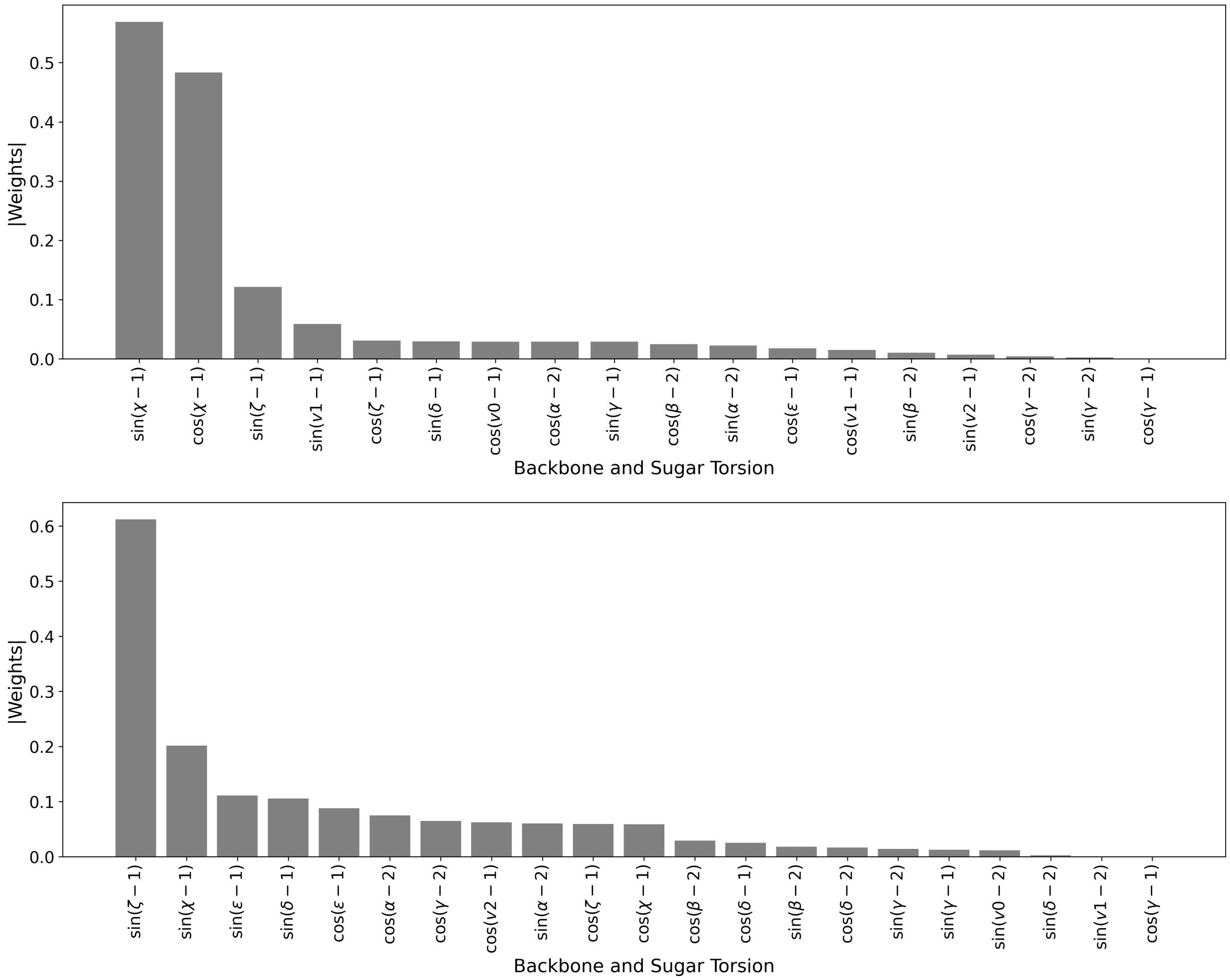

Figure S11: Same as Fig. S5, but for the 2AP unsubstituted C-A dimer.

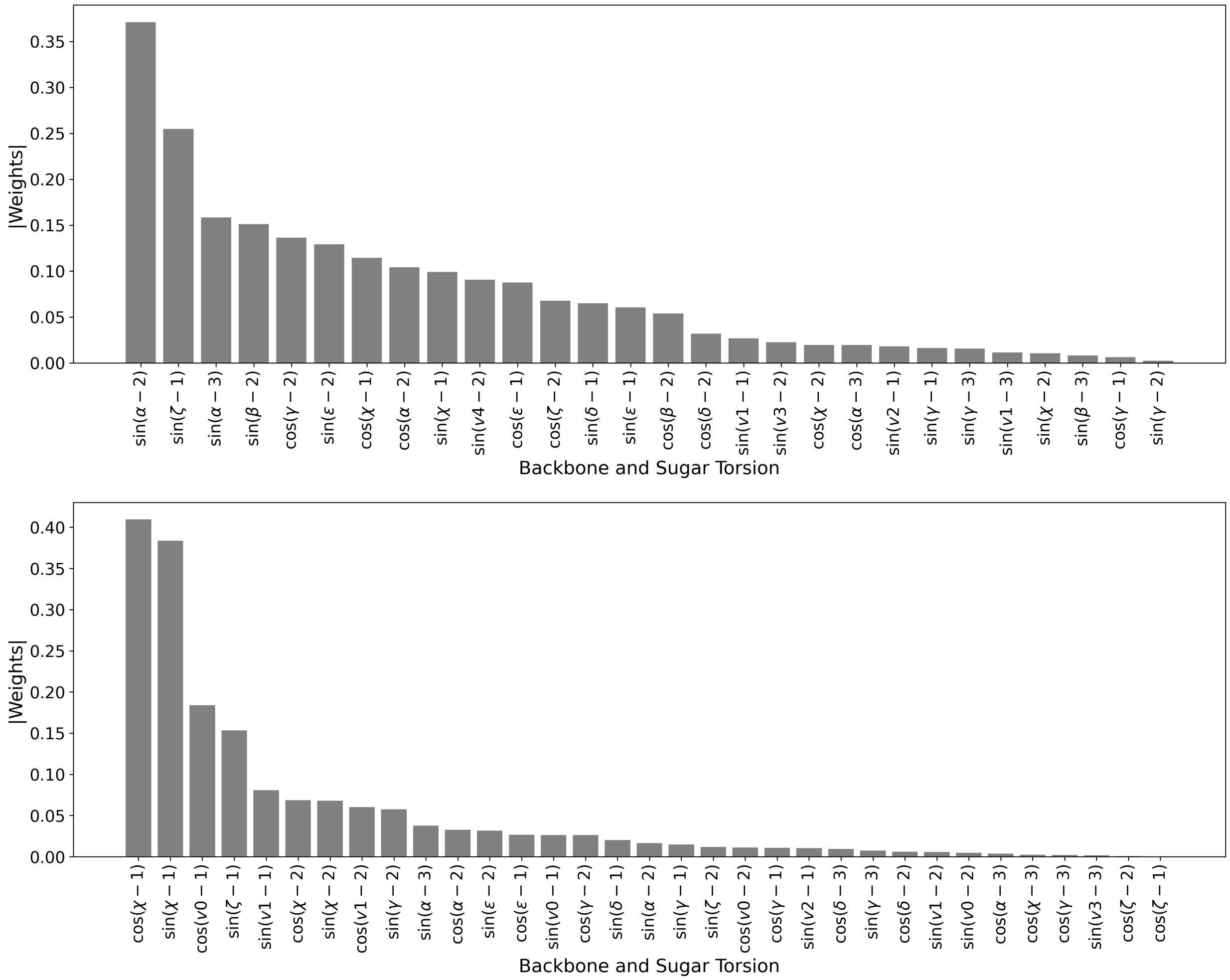

Figure S12: Same as Fig. S5, but for the 2AP unsubstituted C-A-C trimer.

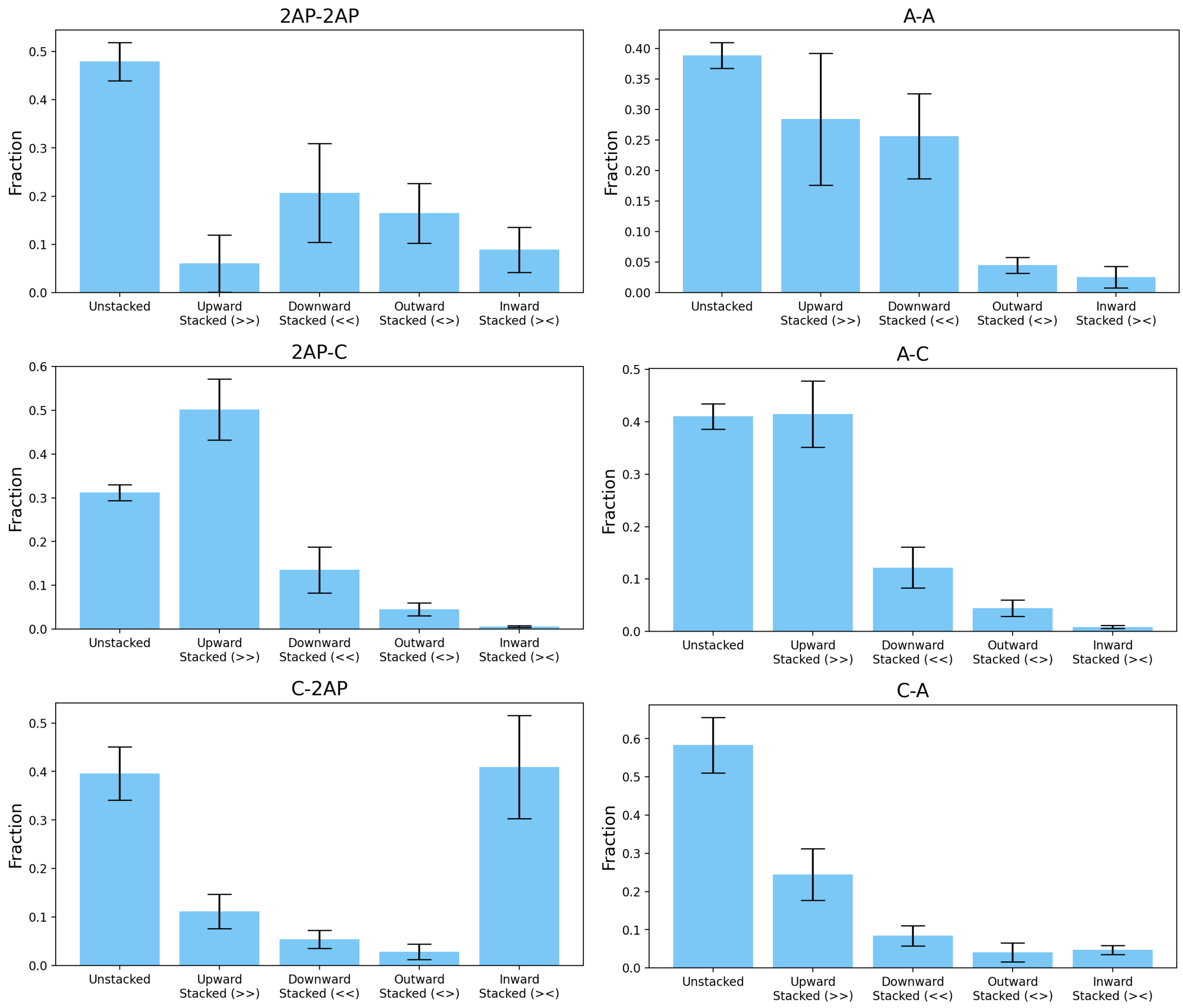

Figure S13: Comparison between the relative populations of the different stacking states of the 2AP substituted (left panel) and unsubstituted (right panel) dinucleotides.

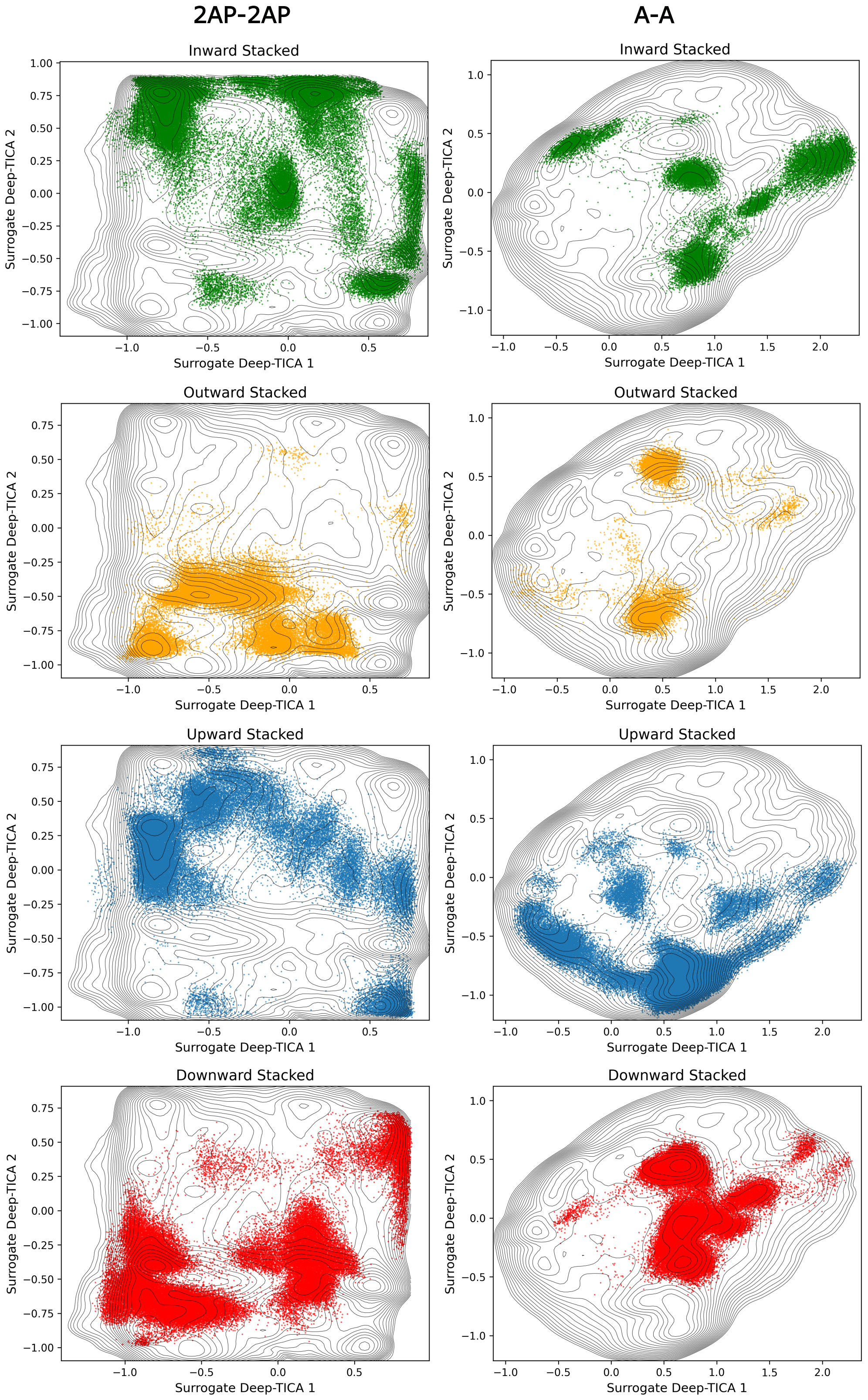

Figure S14: Projection of the different stacking conformers of the 2AP substituted and 2AP unsubstituted A-A dimers projected along the two slowest modes of the Surrogate Deep-TICA CVs.

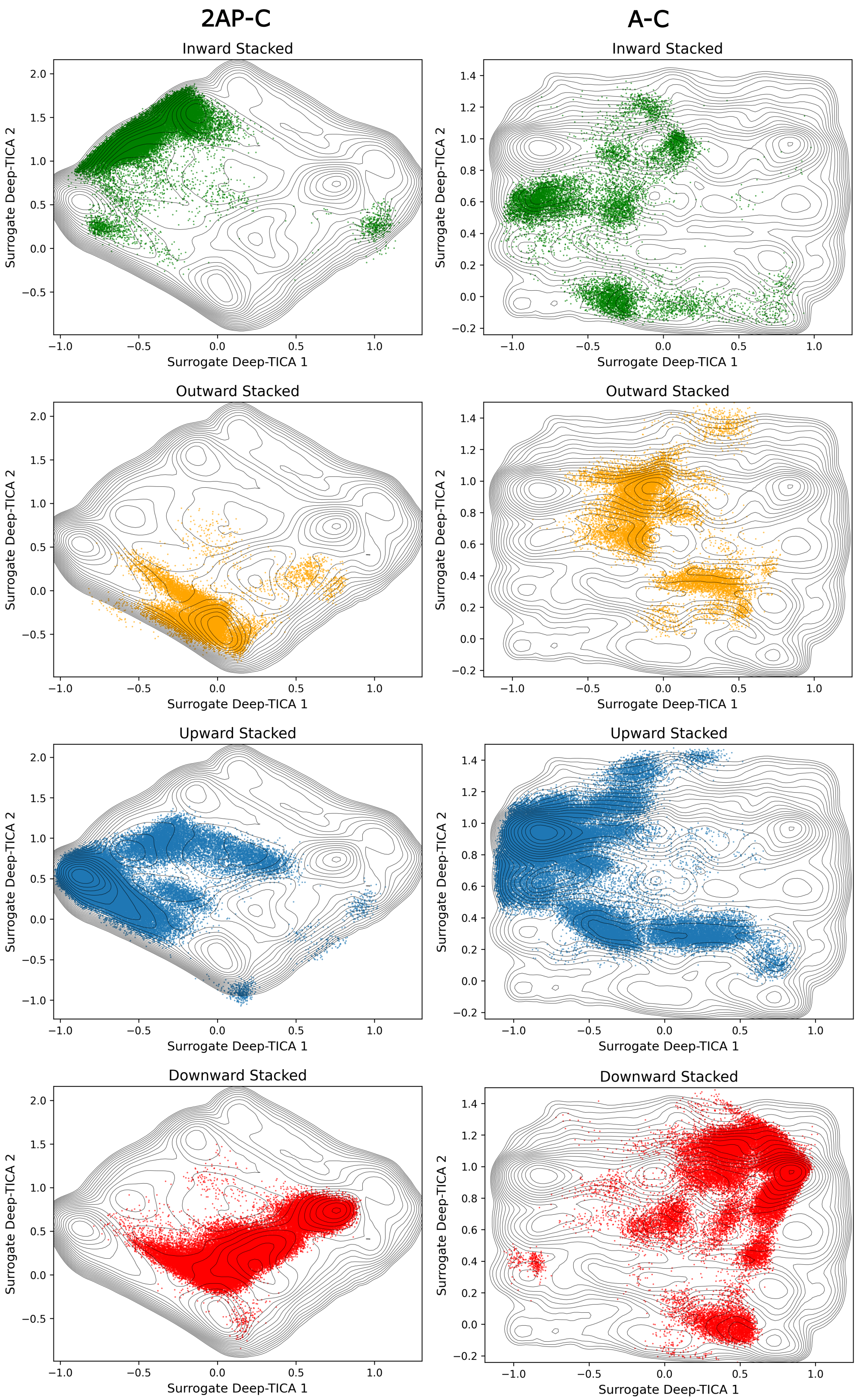

Figure S15: Same as the Fig. S14, but for the 2AP substituted and 2AP unsubstituted A-C dimers.

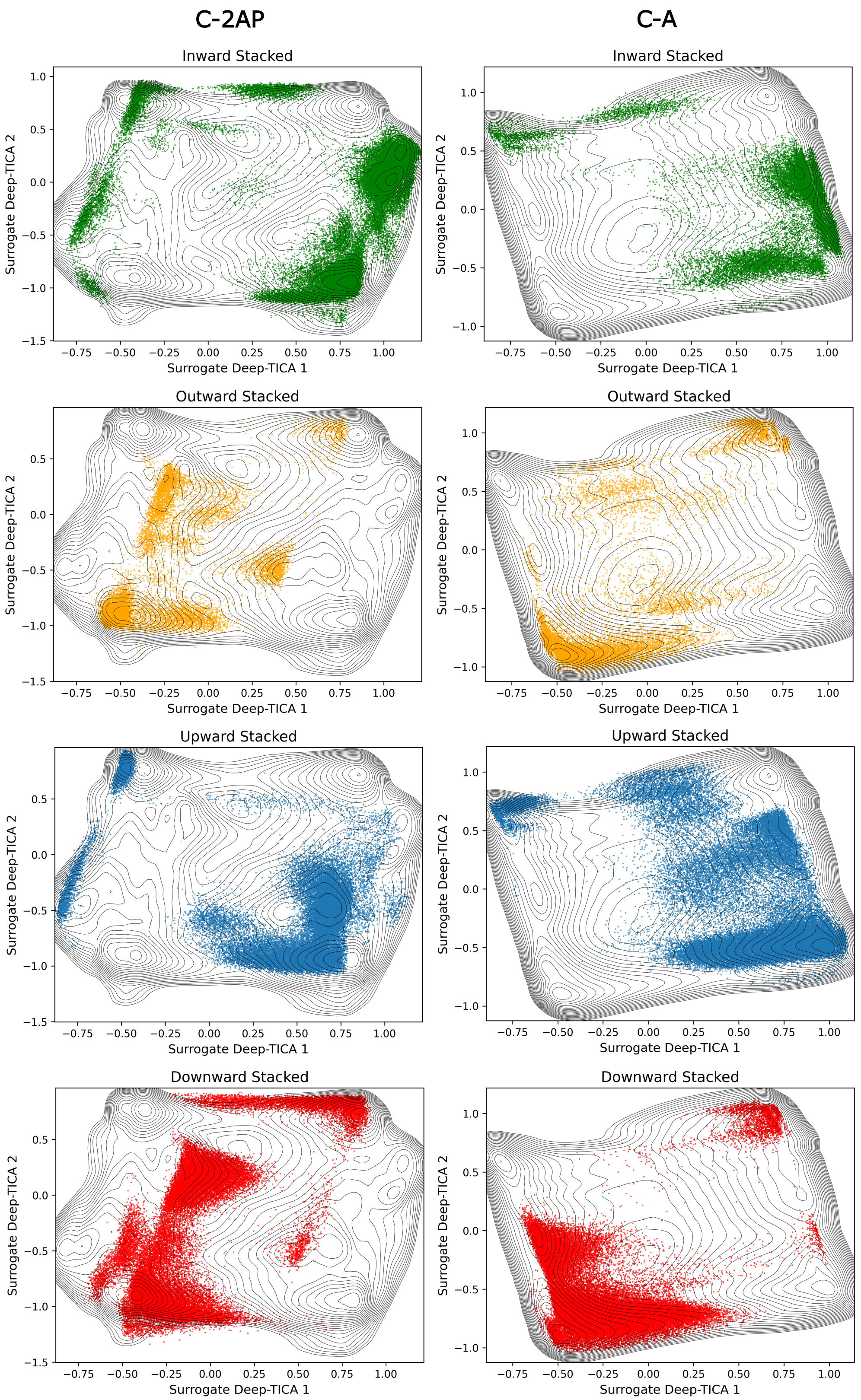

Figure S16: Same as the Fig. S14, but for the 2AP substituted and 2AP unsubstituted C-A dimers.

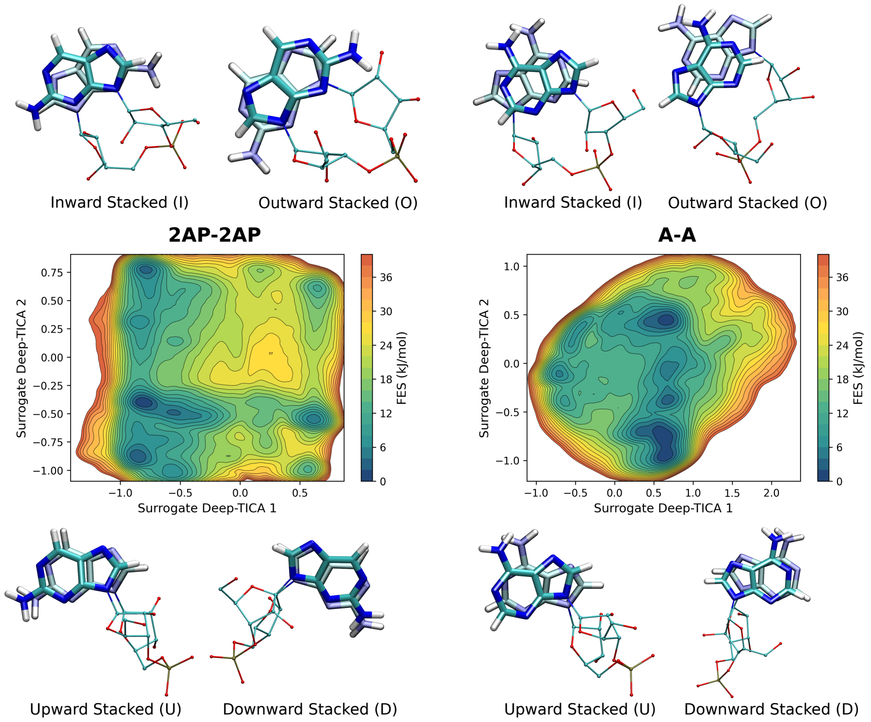

Figure S17: Free energy landscapes of 2AP-substituted and unsubstituted A-A dinucleotides projected along the two slowest modes of the Surrogate Deep-TICA CV. Representative structures for the different stacking modes are provided. The bases are shown in licorice representation, whereas the sugar phosphate backbone is shown in lines.

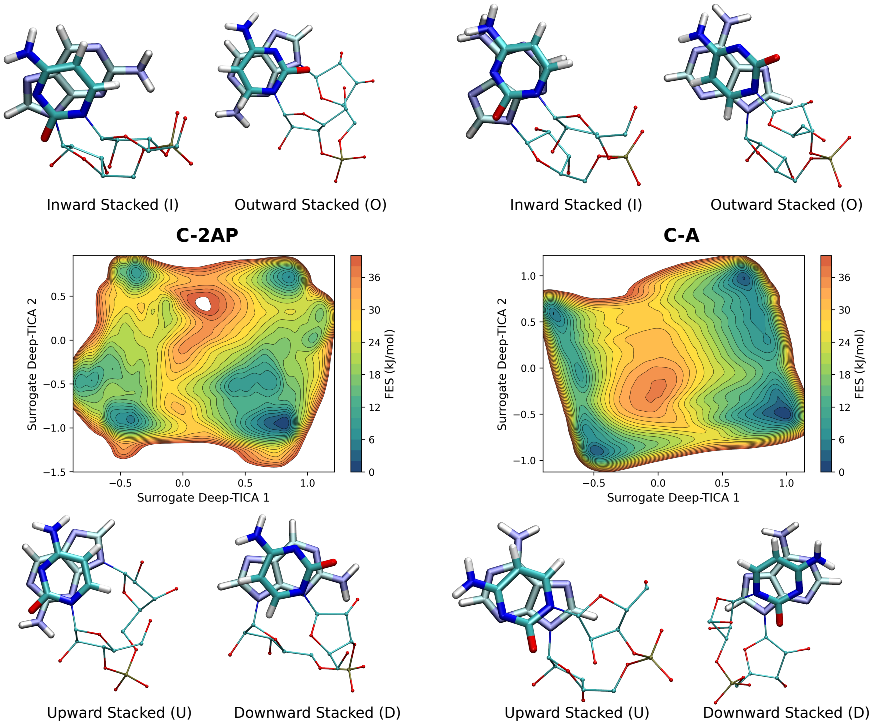

Figure S18: Same as Fig. S17 but for the C-2AP and C-A dimer.

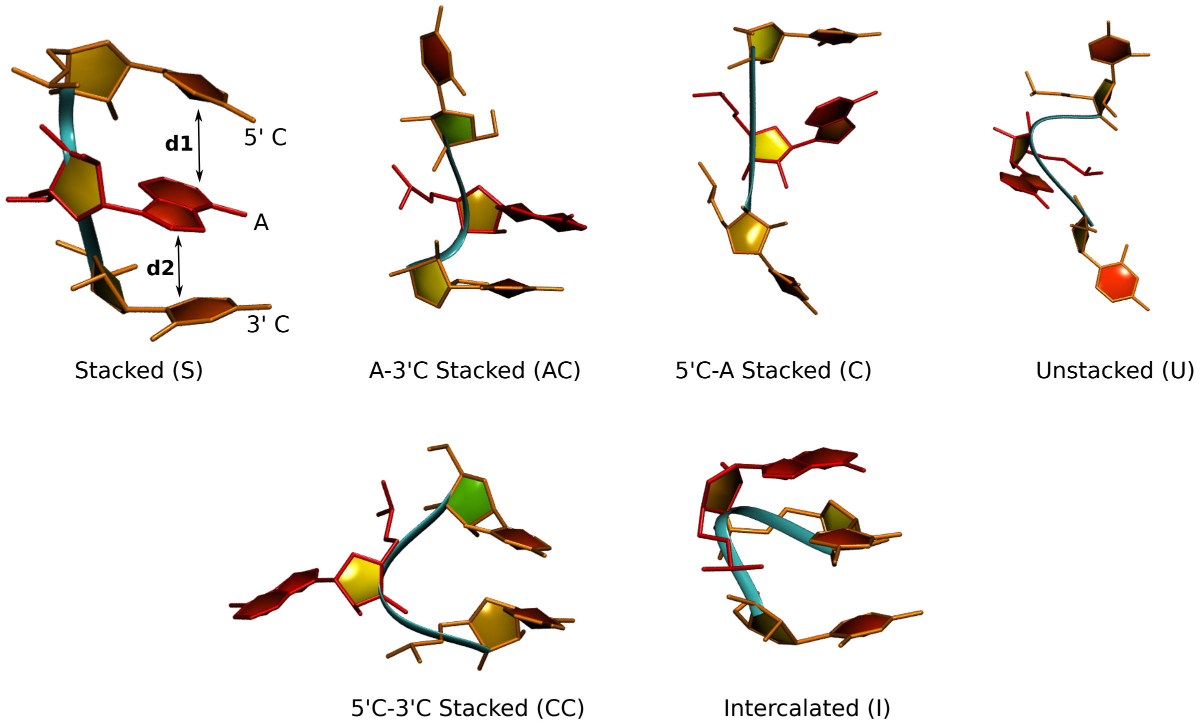

Figure S19: Representative structures of the all stacked, unstacked, and intercalated conformation of the C-A-C trimer.

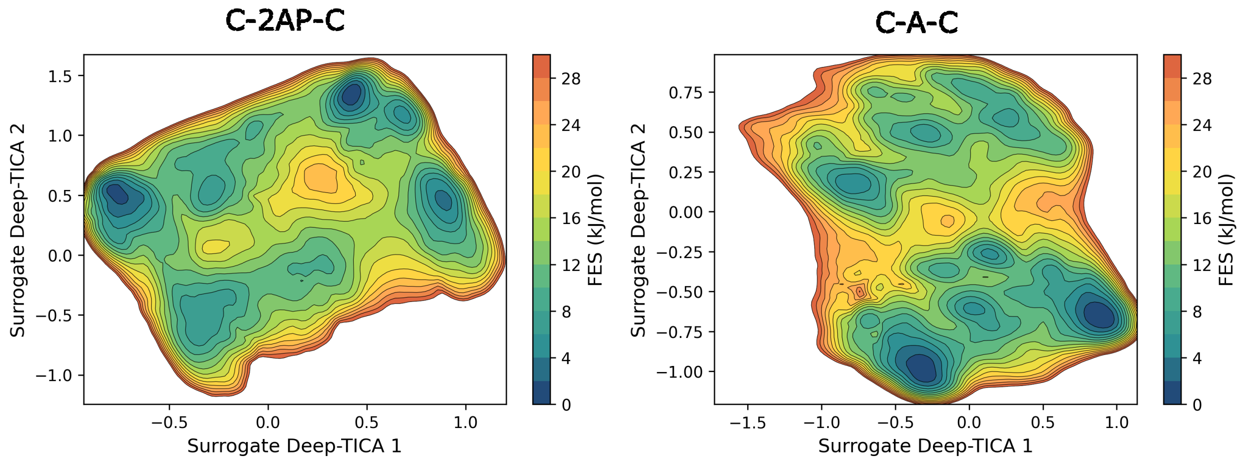

Figure S20: The free energy basins of the 2AP substituted (C-2AP-C) and 2AP unsubstituted (C-A-C) trimers projected along the two modes of the surrogate Deep TICA.

References

(S1) Bonati, L.; Piccini, G.; Parrinello, M. Deep learning the slow modes for rare events sampling. *Proceedings of the National Academy of Sciences* **2021**, *118*, e2113533118.

(S2) Zou, H.; Hastie, T. Regularization and variable selection via the elastic net. *Journal of the Royal Statistical Society Series B: Statistical Methodology* **2005**, *67*, 301–320.

(S3) Bonati, L.; Trizio, E.; Rizzi, A.; Parrinello, M. A unified framework for machine learning collective variables for enhanced sampling simulations: mlcolvar. *The Journal of Chemical Physics* **2023**, *159*.

(S4) Pedregosa, F.; Varoquaux, G.; Gramfort, A.; Michel, V.; Thirion, B.; Grisel, O.; Blondel, M.; Prettenhofer, P.; Weiss, R.; Dubourg, V.; Vanderplas, J.; Passos, A.; Cournapeau, D.; Brucher, M.; Perrot, M.; Duchesnay, E. Scikit-learn: Machine Learning in Python. *Journal of Machine Learning Research* **2011**, *12*, 2825–2830.

(S5) Bottaro, S.; Bussi, G.; Pinamonti, G.; Reißer, S.; Boomsma, W.; Lindorff-Larsen, K. Barnaba: software for analysis of nucleic acid structures and trajectories. *RNA* **2019**, *25*, 219–231.

(S6) Tribello, G. A.; Bonomi, M.; Branduardi, D.; Camilloni, C.; Bussi, G. PLUMED 2: New feathers for an old bird. *Computer physics communications* **2014**, *185*, 604–613.

(S7) Hess, B.; Kutzner, C.; Van Der Spoel, D.; Lindahl, E. GROMACS 4: algorithms for highly efficient, load-balanced, and scalable molecular simulation. *Journal of chemical theory and computation* **2008**, *4*, 435–447.

(S8) Humphrey, W.; Dalke, A.; Schulten, K. VMD – Visual Molecular Dynamics. *Journal of Molecular Graphics* **1996**, *14*, 33–38.
